# *YhjX* Regulates the Growth of *Escherichia coli* in the Presence of a Subinhibitory Concentration of Gentamicin and Mediates the Adaptive Resistance to Gentamicin

**DOI:** 10.3389/fmicb.2019.01180

**Published:** 2019-05-27

**Authors:** Shuqin Zhou, Yijing Zhuang, Xiaojuan Zhu, Fen Yao, Haiyan Li, Huifang Li, Xiaoguang Zou, Jianhua Wu, Huifang Zhou, Gulibaier Nuer, Yuanchun Huang, Shao Li, Qing Peng

**Affiliations:** ^1^Department of Anesthesiology, Zhujiang Hospital, Southern Medical University, Guangzhou, China; ^2^Department of Science and Education, The First Affiliated Hospital of Shantou University Medical College, Shantou, China; ^3^Department of Anesthesiology, First People’s Hospital of Kashi, Kashi, China; ^4^Department of Pharmacology, Shantou University Medical College, Shantou, China; ^5^Department of Pharmacy, First People’s Hospital of Kashi, Kashi, China; ^6^Department of Science and Education, First People’s Hospital of Kashi, Kashi, China; ^7^Department of Clinical Laboratory, First People’s Hospital of Kashi, Kashi, China; ^8^Department of Clinical Laboratory, The First Affiliated Hospital of Shantou University Medical College, Shantou, China; ^9^Department of Hepatobiliary II, Zhujiang Hospital of Southern Medical University, Guangzhou, China

**Keywords:** *Escherichia coli*, adaptive resistance, gentamicin, *YjhX*, bacterial growth

## Abstract

The mechanisms of adaptive resistance of *Escherichia coli* to aminoglycosides remain unclear. Our RNA-Seq study found that expression of *yhjX* was markedly upregulated during initial exposure to subinhibitory concentrations of gentamicin. The expression of *yhjX* was then downregulated dramatically during a second exposure to gentamicin compared to the first exposure. *YhjX* encodes a putative transporter of the major facilitator superfamily, which is known to be the sole target of the YpdA/YpdB two-component system, the expression of which is highly and specifically induced by pyruvate. To investigate the effect of *yhjX* on the adaptive resistance of *E. coli*, in the present study, we constructed *yhjX* deletion and complemented strains of *E. coli* ATCC25922. Changes in extracellular pyruvate levels of wide-type and *yhjX* mutant were measured to determine whether YhjX functions as a pyruvate transporter. The results showed that *yhjX* deletion improved the growth of *E. coli* in medium containing subinhibitory concentrations of gentamicin. The *yhjX* deletion mutant did not exhibit adaptive resistance to subinhibitory concentrations of gentamicin. YhjX might not function as a pyruvate efflux pump in *E. coli* but was associated with the decrease following a sharp increase in the extracellular pyruvate level. Our findings indicate that *yhjX* regulates the growth of *E. coli* in the presence of a subinhibitory concentration of gentamicin and mediates the adaptive resistance to gentamicin.

## Introduction

Aminoglycosides are commonly used to treat clinical infections because of the excellent effects of these compounds against Gram-negative bacteria. However, adaptive resistance to aminoglycosides has been observed *in vitro* and *in vivo* in aerobic and facultative Gram-negative bacilli (Karlowsky et al., 1997b), which might limit the efficacy of these antibiotics in the treatment of clinical infections.

Adaptive resistance to aminoglycosides refers to reduced antimicrobial killing in originally susceptible bacterial populations after initial exposure to aminoglycosides (Karlowsky et al., 1997a; Xiong et al., 1997). Adaptive resistance to aminoglycosides has been reported mostly with *Pseudomonas aeruginosa* but also with *Escherichia coli*, *Staphylococcus aureus*, and other bacteria (Karlowsky et al., 1997b; Motta et al., 2015; Uemura et al., 2017).

Aberrant expression of efflux pumps in the membrane and reduced cellular uptake of aminoglycosides have been commonly considered to be involved in adaptive resistance to aminoglycosides (Hocquet et al., 2003; Skiada et al., 2011). However, the underlying molecular basis of adaptive resistance to aminoglycosides remains unclear. Therefore, it is essential that we gain a better understanding of the causes and mechanisms of adaptive resistance to aminoglycosides.

*Escherichia coli* is a leading pathogen that usually causes infections in the urinary tract and intestines (Katouli, 2010). Although there have been some studies investigating the mechanisms of adaptive resistance of *P. aeruginosa* to aminoglycosides (Gilleland et al., 1989; Daikos et al., 1991; Barclay et al., 1996; Xiong et al., 1997), few studies have examined the adaptive resistance of *E. coli* to aminoglycosides. Previous studies have shown that pretreatment with subinhibitory levels of kanamycin resulted in resistance to subsequent treatment with aminoglycosides in *E. coli* (Sidhu et al., 2012; Xiaocong et al., 2013). In our initial study, we also found that pretreatment with a subinhibitory concentration of gentamicin, another aminoglycoside, induced adaptive resistance to gentamicin in *E. coli* ATCC25922. To investigate the mechanisms involved in this process, we conducted transcriptome sequencing of *E. coli* after pretreatment with subinhibitory concentration of gentamicin. The results of RNA sequencing showed that the expression of *yhjX*, a gene encoding a putative transporter of the major facilitator superfamily, increased 20.65 times compared to that in untreated cells, which was the greatest increase among the upregulated genes. The expression of *yhjX* was then downregulated dramatically during the second exposure to gentamicin compared to the first exposure. This phenomenon suggested that *yhjX* might be involved in the occurrence of adaptive resistance to gentamicin. It encodes a putative major facilitator superfamily transporter with 12 predicted transmembrane helices (Pao et al., 1998). It has been reported that *yhjX* is the sole target of the YpdA/YpdB two-component system, which is strongly and specifically induced by pyruvate (Fried et al., 2013). To investigate the role of *yhjX* in the adaptive resistance of *E. coli* to sub-MIC gentamicin, in this study, we confirmed the changes in expression of *yhjX* in *E. coli* after initial and second exposure to gentamicin and constructed a *yhjX* knockout strain and the corresponding complemented strain. We found that the *yhjX* mutant grew better when exposed to sub-MIC gentamicin initially but less well during the second exposure to gentamicin. It has also been found that when glucuronate or gluconate is present as the primary carbon source, the extracellular pyruvate level increases and *yhjX* expression is induced. Although YhjX protein is annotated as a “putative pyruvate transporter^1^”, this function in *E. coli* has not yet been proven. We suspected that YhjX might be a pyruvate efflux pump that contributes to the slow growth in the presence of gentamicin. To prove this hypothesis, the extracellular pyruvate levels were also measured. However, the extracellular pyruvate levels of the *yhjX*-deleted mutant did not decrease but increased instead in Muller-Hinton broth (MHB) supplemented with gentamicin and M9 minimal medium supplemented with glucuronate compared with those of the wild-type. Our findings demonstrate that *yhjX* regulates the growth of *E. coli* in the presence of a subinhibitory concentration of gentamicin and mediates the adaptive resistance to gentamicin. The protein encoded by *yhjX* is not a pyruvate efflux pump in *E. coli*, and further studies are necessary to investigate the mode of transport and specific substrate of YhjX.

## Materials and Methods

### Bacterial Strain and Determination of the MIC of Gentamicin

*Escherichia coli* strain ATCC25922 was used as the wild-type strain for this study. The MIC of gentamicin was determined using the broth microdilution method recommended by CLSI (Clinical and Laboratory Standards Institute) 2009. Overnight cultures were grown in MHB (Oxoid, United Kingdom, cat:CM0405) at 37°C and diluted to yield an inoculum of approximately 1 × 10^8^ CFU (colony-forming units)/ml. Then, 50 μl of gentamicin (0.5–128 μg/ml) was dispensed into each well of a microtiter plate, and 50 μl of a 10^5^ CFU/ml bacterial suspension was added to each well. The plate was incubated at 37°C for 24 h. The MIC was identified as the lowest concentration of gentamicin at which visible growth was inhibited. Each experiment was replicated three times.

### Determination of Adaptive Resistance by Growth Curve Analysis

A single colony of *E. coli* ATCC 25922 was inoculated in 5 ml of MHB and incubated overnight at 37°C with shaking at 200 rpm. The overnight bacterial culture was diluted 1:20 in fresh MHB pretreated with 1 μg/ml (1/2 MIC) gentamicin at 200 rpm for 1 h at 37°C. The pretreated culture was then centrifuged at 10,000 rpm for 3 min at room temperature, and the pellet was washed 3 times with fresh media and then resuspended in MHB. The bacterial suspension was adjusted to a final OD600 of 0.2 (as detected by a Bio-Rad spectrophotometer). Simultaneously, the non-pretreated cultures were also centrifuged and resuspended as described above. The bacterial suspensions were diluted 1:1 with MHB containing gentamicin. The final concentration of gentamicin in each suspension was 1 μg/ml (1/2 MIC). Then, 100 μl of each suspension containing gentamicin was added to a 96-well plate and placed in a microplate reader (Molecular Devices, SpectraMax M2e) for monitoring of bacterial growth at 600 nm. Fifty microliters of sterile paraffin oil was added into each well to avoid fluid evaporation. Readings were taken every 30 min for 24 h by the microplate reader. Each experiment was performed in triplicate.

### RNA Extraction, Sequencing and Analysis

Overnight cultures were diluted 1:20 and then either treated with 1/2 MIC gentamicin or left untreated and incubated at 37°C for 1 h. The cells were harvested for RNA isolation. Total RNA was isolated using the RNeasy Protect Mini Kit (Qiagen, Hilden, Germany, cat.: 74134) according to the manufacturer’s instructions. RNA sequencing (RNA-Seq) was performed by Shanghai Bohao Co., Ltd., using an Illumina HiSeq 2500 (Illumina) as previously described (Li et al., 2016). RNA integrity and purity were analyzed using an Agilent 2100 bioanalyzer. The transcriptome sequencing data were aligned with the genome and plasmid sequences of *E. coli* ATCC 25922 (GenBank: CP009072.1 and CP009073.1) in the NCBI database. The relative gene expression levels were estimated by RPKM (reads per kilobase of exon sequence per million mapped reads) for normalization of gene expression (Heo et al., 2014).

### Quantitative Real-Time PCR

Quantitative real-time PCR (qRT-PCR) was performed to verify the results of RNA-Seq. The RNA was converted to cDNA (Takara, Dalian, China, cat.: RR047A) by reverse transcription. One microliter of cDNA was amplified (Takara, cat.: RR820A) using an ABI 7500 real-time PCR system. *YhjX* and its regulator gene *ypdB*, *fliN* (flagellar motor switch protein), *cpxP* (inhibitor of the cpx response periplasmic adaptor protein), *gltA* (type II citrate synthase), *aceE* (pyruvate dehydrogenase), *sdhC* and *sdhD* (succinate dehydrogenase cytochrome b556 small membrane subunits) were selected for real-time PCR studies. The GAPDH gene was used as the housekeeping reference gene (Lee et al., 2010). The primers used for real-time PCR quantification of the expression of each gene are listed in Table 1. The fold change was calculated using the 2ΔΔCt method and is presented as the fold change in the expression of pretreatment groups relative to that of the control group (no drug treatment). In addition, the fold change of most highly upregulated gene in RNA-Seq, namely, *yhjX*, and its regulator *ypdB* were also detected in cells that were re-exposed to a sub-MIC of gentamicin.

**Table 1:** Primers used for qRT-PCR.

Gene	Forward primer	Reverse primer	References
*yhjX*	ATGTATGTGATTGGTGTAGCGAAAG	CAGAAAGGTTGGCGATGGA	
*ypdB*	CATTACCGGGATGCTGCAAA	GGTCATTTTCTCGTGCGCTT	
*fliN*	GGACGATCTGTGGGCTGAA	GACATCACCACCGCCAAA	
*cpxP*	GGCATCCGGGTGAAGAACTT	AACTTATGCCGTCGAACATATGG	Price and Raivio, 2009
*gltA*	AACTTATGCCGTCGAACATATGG	TGTTTTCCAGCTCCATAGCC	Martinez et al., 2010
*aceE*	ACGTACCGGCTGACGACTAC	CTTATCGATTTCGCCACGTT	Lin et al., 2005
*sdhC*	AGATTTTGGCGGAGCGTTT	ATTTATCATGTGGGGCATCCT’	
*sdhD*	CGGTCAACACCTGCCACAT	TGGATTGGTTTCTTCGCCTCT	
*GAPDH*	ACTTACGAGCAGATCAAAGC	AGTTTCACGAAGTTGTCGTT	

### Construction of the Δ*yhjX* Mutant and Complemented Strain

The Δ*yhjX* mutant was constructed using the suicide T-vector pLP12 carrying a counterselectable marker, vmi480 (Luo et al., 2015). Briefly, a *yhjX* gene fusion fragment was amplified by PCR, ligated with pLP12 and subsequently transformed into *E. coli* β2163. The resulting plasmids were introduced into *E. coli* ATCC25922 via conjugation with *E. coli* β2163. After two rounds of selection, the mutant with the *yhjX* gene deleted was validated by PCR using primers corresponding to sequences upstream and downstream of the deletion and by subsequent sequencing. The *yhjX* knock-out mutant was transformed with the pBAD30:*yhjX* plasmid (carrying ampicillin resistance marker gene ampR) to obtain the *yhjX*-complemented strain. The complemented strain was cultured in medium containing 100 μg/ml ampicillin. L-arabinose (0.2%) was added to the medium to induce the expression of *yhjX* in the complemented strain.

### Determination of the MICs of Gentamicin and Other Antibiotics Against the ATCC 25922 Wild-Type, Δ*yhjX* Mutant and Complemented Strains

The MICs of gentamicin and other antibiotics, including cefuroxime, cefotiam, ceftazidime, ciprofloxacin, and imipenem, against the ATCC 25922 wild-type, Δ*yhjX* mutant and complemented strains were determined using the broth microdilution method as previously mentioned.

### Growth Curve of the *E. coli* Wild-Type, Δ*yhjX* Mutant and Complemented Strains in the Presence and Absence of a Subinhibitory Concentration of Gentamicin

Overnight cultures of the *E. coli* ATCC25922 wild-type, Δ*yhjX* mutant and complemented strains were diluted in fresh MHB to a final OD600 of 0.2. The suspensions were diluted 1:1 with MHB with or without gentamicin in a 96-well plate. The treated cells were grown in the presence of 1/2 MIC gentamicin, and the control was treated with MHB. Then, 50 μl of sterile paraffin oil was added into each well to avoid fluid evaporation. The 96-well plate was then placed into a microplate reader for OD600 measurements every 0.5 h at 37°C for 24 h. The experiment was replicated three times.

### Determination of Adaptive Resistance of the *E. coli* Wild-Type, Δ*yhjX* Mutant and Complemented Strains by Growth Curve Analysis

The same method used for the adaptive resistance experiment above was used to examine the *E. coli* wild-type, Δ*yhjX* mutant and complemented strains.

### Measurement of Extracellular Pyruvate Levels in *E. coli*Δ*yhjX* Mutant and Wild-Type Cultures and Relative Expression of *yhjx* in *E. coli* Grown in Different Media

Overnight cultures of the ATCC 25922 wild-type and Δ*yhjX* mutant strains were diluted with fresh media (MHB with or without ½ MIC gentamicin, M9 minimal medium containing 0.4% glucuronate and M9 minimal medium containing 0.4% glucose) to a final OD600 of 0.2. The levels of pyruvate in fresh culture and in supernatants of *E. coli* cultures were determined before inoculation, 30 min after inoculation and 60 min after inoculation using a pyruvate colorimetric/fluorometric assay kit (BioVision). Each experiment was replicated three times. The experimental values were calculated from a standard curve.

Quantitative RT-PCR was also performed to compare the expression levels of *yhjX* in the wild-type *E. coli* strain in different media and to analyze the relationship between *yhjX* expression levels and extracellular pyruvate concentrations. The *yhjX* expression in *E. coli* growing in M9 containing glucose at the 30 min time point was set as the control.

## Results

### Adaptive Resistance Detected in *E. coli* After Pretreatment With a Subinhibitory Concentration of Gentamicin

**FIGURE 1:**
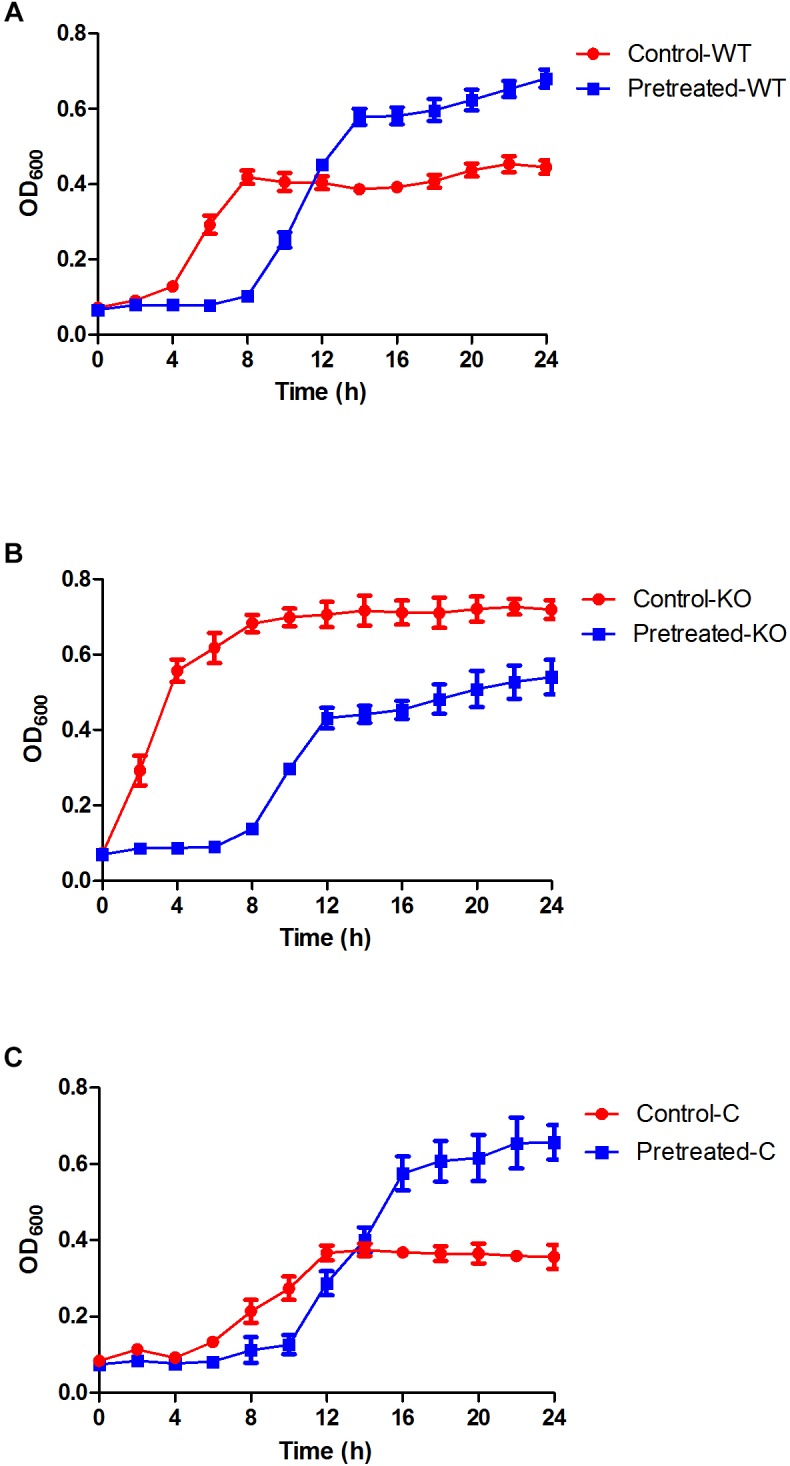
Growth curves of the *E. coli* ATCC 25922 wild-type, Δ*yhjX* mutant and complemented strains in the presence of subinhibitory concentrations of gentamicin. **(A)** Control-WT: growth curve of *E. coli* ATCC25922 wild-type (without pretreatment) in MHB with ½ MIC gentamicin; pretreated-WT: growth curve of *E. coli* ATCC25922 wild-type (pretreated with ½ MIC gentamicin) in MHB with ½ MIC gentamicin. **(B)** Control-KO: growth curve of the Δ*yhjX* knock-out strain (without pretreatment) in MHB with ½ MIC gentamicin; pretreated-KO: growth curve of the Δ*yhjX* knock-out strain (pretreated with ½ MIC gentamicin) in MHB containing ½ MIC gentamicin. **(C)** Control-C: growth curve of the Δ*yhjX* complemented strain (without pretreatment) in MHB with ½ MIC gentamicin; pretreated-C: growth curve of the Δ*yhjX* complemented strain (pretreated with ½ MIC gentamicin) in MHB containing ½ MIC gentamicin.

The MIC of *E. coli* 25922 against gentamicin was 2.0 μg/ml. As shown in Figure 1A, in comparison to the control (exposed to gentamicin for the first time), *E. coli* pretreated with ½ MIC gentamicin for a short duration (1 h) exhibited low growth during the early phase (8 h of re-exposure to gentamicin), suggesting a postantibiotic effect (PAE) caused by gentamicin. However, the growth rate of the pretreated cells markedly increased during the late phase (from 10 to 24 h, *p* = 0.027 at the 24-h time point). This result suggested that the adaptive resistance of *E. coli* could be induced by initial exposure and re-exposure to subinhibitory concentrations of gentamicin.

### Comparative Transcriptomic Analysis of *E. coli* ATCC 25922 Exposed to a Sub-MIC of Gentamicin

To screen out genes that may be involved in the development of adaptive resistance, RNA-Seq of untreated *E. coli* and *E. coli* treated with a sub-MIC of gentamicin was performed. We selected genes that showed a twofold change in expression after treatment with sub-MIC gentamicin compared with the expression in control cells that were not exposed to gentamicin. In response to gentamicin, the expression levels of 235 genes were upregulated (Supplementary Table S1), and the levels of 349 genes were downregulated (Supplementary Table S2). The roles of differentially regulated genes were assigned according to the KEGG database^2^. The results were verified using qRT-PCR (Table 2).

**Table 2:** Validation of RNA-Seq results using qRT-PCR.

Gene	Description	RNA-Seq	qRT- PCR
		
		Fold change	
*yhjX*	Membrane protein	20.65	46.57
*fliN*	Flagellar motor switch protein	2.18	5.04
*cpxP*	Inhibitor of the cpx response periplasmic adaptor protein	11.26	23.74
*gltA*	Type II citrate synthase	0.32	0.24
*aceE*	Pyruvate dehydrogenase	0.49	0.34
*sdhC*	Succinate dehydrogenase cytochrome b556 small membrane subunit	0.43	0.31
*sdhD*	Succinate dehydrogenase cytochrome b556 small membrane subunit	0.47	0.25
*ypdB*	Regulator of yhjX	0.75	0.89

**FIGURE 2:**
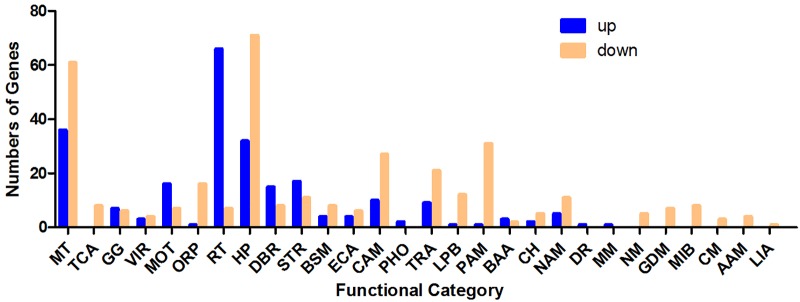
Number of genes (classified by functional category) upregulated and downregulated in response to sub-MIC gentamicin in the RNA-Seq data. MT, membrane and transporter; TCA, TCA cycle; GG, glycolysis/gluconeogenesis. VIR, virulence; MOT, motility; ORP, oxidation–reduction process; RT, ribosome and translation; HP, hypothetical protein; DBR, DNA binding and recombination; STR, stress response; BSM, biosynthesis of secondary metabolites; ECA, electron carrier activity; CAM, other carbohydrate metabolism processes; PHO, phosphorylation; TRA, transcription; LPB, lipid biosynthesis; PAM, protein and amino acid metabolism; BAA, biosynthesis of amino acids; CH, cell shape; NAM, nucleic acid metabolism; DR, DNA replication; MM, methane metabolism; NM, nitrogen metabolism; GDM, glyoxylate and dicarboxylate metabolism; MIB, metal ion binding; CM, carnitine metabolic process; AAM, ascorbate and aldarate metabolism; LIA, lysozyme inhibitor activity.

As shown in Figure 2, differentially regulated genes were mainly enriched in the categories membrane and transporter, ribosome and translation, stress response, motility, TCA (tricarboxylic acid) cycle, glycolysis/gluconeogenesis and other carbohydrate metabolism processes, protein and amino acid metabolism, transcription, DNA binding and recombination, nucleic acid metabolism, oxidation–reduction process and hypothetical proteins with unknown functions. Ninety-seven genes with membrane and transporter functions were differentially expressed; 36 of these genes were upregulated, and 61 were downregulated. *YhjX*, a gene encoding a putative transporter of the major facilitator superfamily, was upregulated with a 20.65-fold change in expression, which was the highest fold change among all the genes. Sixty-six genes involved in ribosome and translation were upregulated, and 7 such genes were downregulated. A total of 56 genes involved in the TCA cycle, glycolysis/gluconeogenesis and other carbohydrate metabolism were also differentially expressed. Twenty-eight genes involved in stress response and 22 genes involved in motility were differentially regulated.

### *YhjX* Was Highly Activated When Exposed to Subinhibitory Concentrations of Gentamicin

**FIGURE 3:**
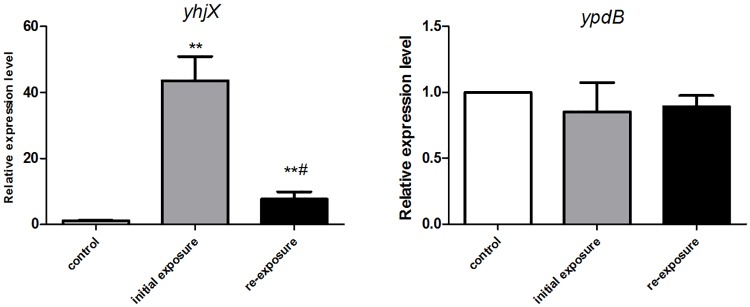
Changes in gene expression of *yhjX* and *ypdB* in *E. coli* ATCC25922 wild-type after initial exposure and re-exposure to ½ MIC gentamicin. ^∗∗^ indicates a statistically significant difference (*P* < 0.01) compared to the control (without treatment); ^#^ indicates a statistically significant difference (*P* < 0.01) compared to initial exposure to gentamicin.

YpdB protein has been proven to be the regulator of *yhjX*, functioning by binding to two direct repeats of a motif in the *yhjX* promoter. We therefore performed qRT-PCR to detect the differential expression of *yhjX* and *ypdB* in *E. coli* initially exposed to and then re-exposed to subinhibitory concentrations of gentamicin. An untreated strain was set as the control group. As shown in Figure 3, compared to the untreated cells, *yhjX* of *E. coli* was 43.6- and 7.6-fold upregulated after first exposure and second exposure to ½ MIC gentamicin, respectively. However, the expression level of *ypdB* was unchanged after both initial exposure and re-exposure to gentamicin.

### *YhjX* Deletion Did Not Influence the MICs of Gentamicin and Other Antibiotics

To understand whether the expression of *yhjX* affects the sensitivity of *E. coli* to antibiotics, we determined the MICs of gentamicin and other antibiotics, including cefuroxime, cefotiam, ceftazidime, ciprofloxacin, and imipenem, against the ATCC 25922 wild-type, Δ*yhjX* mutant and complemented strains. The MICs of gentamicin, cefuroxime, cefotiam, ceftazidime, and imipenem against ATCC 25922 wild-type were 2, 4, 0.25, 0.25, and 0.25 μg/ml, respectively. Ciprofloxacin showed the strongest antibacterial effect against ATCC 25922 (MIC ≤ 0.0625). The MICs of all the tested antibiotics against the Δ*yhjX* mutant were the same as those against the wild-type, suggesting that *yhjX* deletion did not influence the MICs of gentamicin and other antibiotics. The MICs of cefotiam and ceftazidime against the complemented strain increased to 1 μg/ml from the original value of 0.25 μg/ml against the wild-type. Except for the increase in the MICs of cefuroxime and ceftazidime, there was no change in the MICs of the antibiotics against the complemented strain. However, as the vector in the complemented strain carries the ampicillin resistance gene ampR, the changes in the MICs of cefuroxime and ceftazidime might not be associated with the overexpression of *yhjX*.

### *YhjX* Deletion Improved the Growth of *E. coli* in Medium Containing a Subinhibitory Concentration of Gentamicin

**FIGURE 4:**
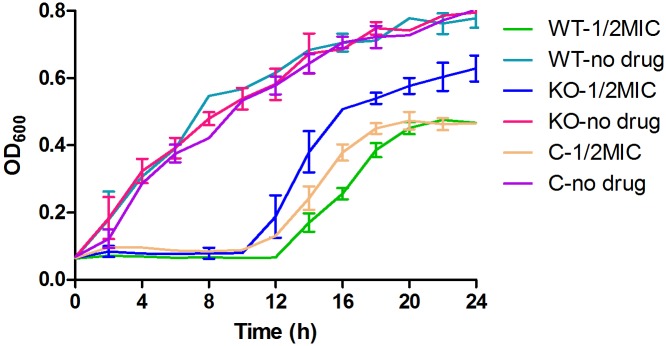
Growth curves of the *E. coli* wild-type, Δ*yhjX* mutant and complemented strains in the presence or absence of ½ MIC gentamicin. WT-1/2MIC, growth curve of *E. coli* ATCC25922 wild-type in MHB containing ½ MIC gentamicin; WT-no drug, growth curve of *E. coli* ATCC25922 wild-type in MHB with no drug; KO-1/2MIC, growth curve of the *E. coli* ATCC25922 Δ*yhjX* mutant in MHB containing ½ MIC gentamicin; KO-no drug, growth curve of the *E. coli* ATCC25922 Δ*yhjX* mutant in MHB with no drug; C-1/2MIC, growth curve of the *E. coli* ATCC25922 Δ*yhjX* complemented strain in MHB containing ½ MIC gentamicin; C-no drug, growth curve of the *E. coli* ATCC25922 Δ*yhjX* complemented strain in MHB with no drug.

When cultured in MH medium, the growth curves of the Δ*yhjX* mutant and complemented strain were very similar to that of the wild-type *E. coli*. Although a change in MIC was not observed among these strains, the Δ*yhjX* mutant exhibited a higher growth rate in the ½ MIC gentamicin-containing medium that the wild-type and complemented strains (Figure 4). As shown in Figure 3, both the wild-type and complemented strains cultured in ½ MIC gentamicin reached stationary phase at 18 h, while the Δ*yhjX* mutant remained in the late exponential growth phase.

### The Δ*yhjX* Mutant Did Not Exhibit Adaptive Resistance to a Subinhibitory Concentration of Gentamicin

After pretreatment with ½ MIC gentamicin for 1 h, the Δ*yhjX* mutant was washed and re-exposed to ½ MIC gentamicin but showed lower growth than the mutant without pretreatment (Figure 1B,C). This result indicated that the *yhjX*-deleted mutant did not exhibit adaptive resistance to subinhibitory concentrations of gentamicin, unlike the wild-type strain.

### YhjX Was Not a Pyruvate Efflux Pump but Was Associated With the Decrease Following an Increase in Extracellular Pyruvate Levels

A previous study demonstrated that extracellular pyruvate stimulated the induction of *yhjX*. *YhjX* induction was observed in LB medium and M9 minimal medium with gluconate or glucuronate (Fried et al., 2013). When glucose was the sole C source, extracellular pyruvate levels did not increase, and the expression levels of *yhjX* remained low. To verify these results and compare the expression of *yhjX* in different media, the extracellular concentrations of pyruvate were determined, and relative *yhjX* expression was detected by qRT-PCR.

**FIGURE 5:**
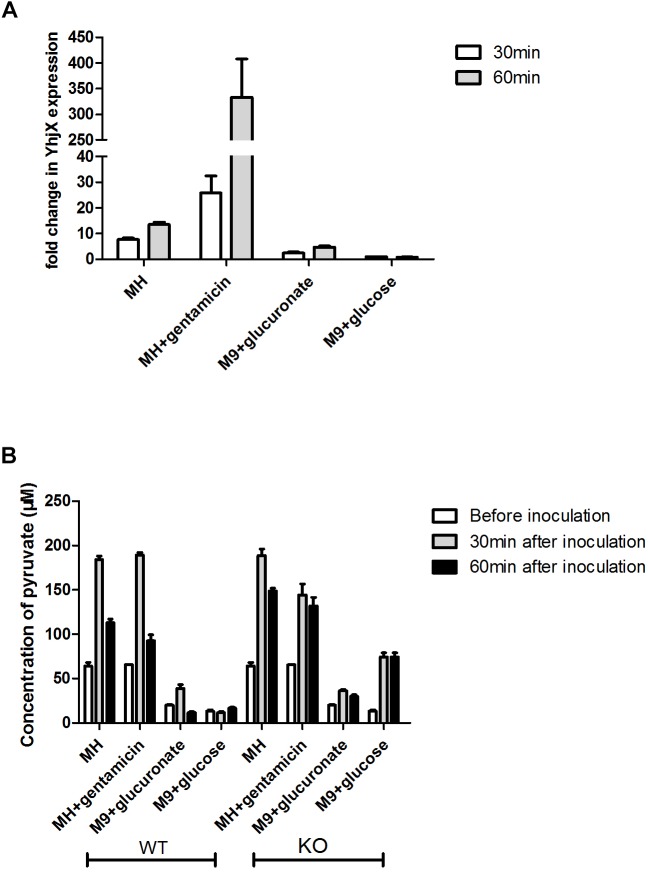
Comparison of relative expression of yhjX and extracellular pyruvate measurement in different media. **(A)** Relative expression of yhjX in *E. coli* ATCC 25922 wild-type in different media, including MHB, MH medium with ½ MIC gentamicin, M9 minimal medium containing 0.4% glucuronate and M9 minimal medium with 0.4% glucose. **(B)** ATCC 25922 wild-type (WT) and ΔyhjX knock-out mutant (KO) were inoculated in MHB with and without ½ MIC gentamicin, M9 containing 0.4% glucuronate and M9 containing 0.4% glucose. Concentrations of extracellular pyruvate in different cultures were determined before inoculation, 30 min after inoculation and 60 min after inoculation.

As shown in Figure 5A, the expression levels of *yhjX* in *E. coli* grown in MH medium, MH medium with gentamicin and M9 minimal medium showed a higher fold change at both 30 and 60 min than the expression levels in the control (grown in M9 minimal medium with glucose). The expression level of *yhjX* in *E. coli* grown in MH medium with gentamicin was dramatically upregulated compared to that in other media.

Figure 5B shows that the basal concentrations of pyruvate in MH medium and MH plus gentamicin were more than 3 times higher than those in M9 medium containing glucuronate or glucose. A sharp increase in extracellular pyruvate levels at 30 min and a subsequent more than 40% decrease at 60 min could be detected when *E. coli* wild-type was grown in MH medium, MHB containing 0.4% gentamicin and M9 containing 0.4% glucuronate. There were no significant changes in pyruvate concentration at different time points for the wild-type grown in M9 medium plus glucose. In contrast, for the *yhjX* mutant, no significant decrease (*p* > 0.05) was detected at 60 min after inoculation (compared to the level of pyruvate at 30 min) in any of the media. In addition, the extracellular levels of pyruvate of the *yhjX* knock-out mutant were higher at 60 min after inoculation than those of the wild-type strain grown in all four media. These results suggested that YhjX might not play the role of pyruvate efflux pump in *E. coli* but was associated with the decrease following a sharp increase in the extracellular pyruvate level.

## Discussion

*In vitro*, animal and clinical studies have shown that the development of marked adaptive resistance of Gram-negative bacteria to aminoglycosides occurs within 1–2 h of the first dose. Adaptive resistance to aminoglycosides seems to be caused not by a genetic mutational change but rather by a protective phenotypic alteration of bacteria. It has been reported that exposure of bacteria to sublethal concentrations of antibiotics can lead to increased efflux pump expression, providing adaptive antibiotic resistance (Sidhu et al., 2012). However, the mechanisms of adaptive resistance of Gram-negative bacteria to aminoglycosides remain unclear.

Adaptive resistance could defined as reduced antimicrobial susceptibility in bacteria after initial exposure to antibiotics. This study confirmed that the adaptive resistance of *E. coli* ATCC 25922 could be induced by initial exposure to subinhibitory concentrations of gentamicin. To improve the understanding of the molecular mechanisms involved in adaptive resistance in *E. coli*, we performed whole-transcriptome profiling of ATCC 25922 using RNA-Seq. RNA-Seq data (Supplementary Tables S1, S2) showed that a high number of genes associated with membrane and transporter functions were strongly regulated, suggesting that these genes might play key roles in the gentamicin tolerance of *E. coli.* Sixty-six genes associated with ribosome and translation were upregulated, and 7 such genes were downregulated. We hypothesize that the changes in the expression of these genes are associated with the mechanism of action of gentamicin, which inhibits protein synthesis by binding to the 30S subunit of the ribosome. A total of 56 genes involved in the TCA cycle, glycolysis/gluconeogenesis and other carbohydrate metabolism processes were also differentially expressed. Notably, sub-MICs of gentamicin clearly inhibit the TCA cycle of *E. coli* by downregulating 8 genes involved in the TCA cycle. These genes include the pyruvate dehydrogenase-encoding genes DR76_21695 and *aceE*; type II citrate synthase-encoding gene *gltA*; *sdhD* and *sdhC*, encoding succinate dehydrogenase cytochrome b556 small membrane subunits; *sucC*, encoding succinyl-CoA synthetase subunit beta; the isocitrate dehydrogenase-encoding gene DR76_19495; and the fumarate hydratase class I-encoding gene DR76_16820. These genes encode the key citric acid cycle enzymes that contribute to energy production and metabolism. We speculate that TCA cycle inhibition and thereby the scarcity of energy sources caused by gentamicin might be the reason underlying the altered expression of genes involved in glycolysis/gluconeogenesis and other carbohydrate metabolism pathways. RNA-Seq also showed that 22 genes associated with bacterial motility were differentially regulated. Among these genes, 16 genes associated with flagella and fimbriae were upregulated. Our previous study proved that pretreatment with subinhibitory concentrations of gentamicin inhibits the swarming motility of *E. coli* ATCC 25922 (Zhuang et al., 2016), suggesting that high expression of these flagella- and fimbriae-associated genes was more likely a stress-related feedback of *E. coli* in response to the limited energy supply. Twenty-eight stress response genes were also differentially regulated by treatment with a sub-MIC of gentamicin. These genes may play a role in protecting *E. coli* from damage caused by environmental stress.

Our transcriptome sequencing data showed that *yhjX*, encoding a putative protein that is a transporter of the major facilitator superfamily (Behr et al., 2014), was the most highly upregulated gene during the first exposure to a subinhibitory concentration of gentamicin. The expression of *yhjX* in *E. coli* was then confirmed by qRT-PCR. The results showed that *yhjX* expression was upregulated 46.6- and 7.2-fold in during initial exposure and re-exposure to ½ MIC gentamicin, respectively, compared to that in untreated cells. These data suggest that *yhjX* is a sensitive gentamicin response-related gene in *E. coli*.

The changes in expression of *yhjX* between the first and second exposures to gentamicin seem to be associated with the growth status of *E. coli* in the presence of gentamicin. To investigate the involvement of the *yhjX* gene in the growth of *E. coli* and the tolerance of the cells to gentamicin, we constructed the *yhjX*-deleted strain and the complemented strain. We observed that there was no difference in growth rate among the wild-type, *yhjX*-deleted and complemented strains cultured in MH medium. In addition, *yhjX* deletion did not influence the MICs of gentamicin or other antibiotics, including cefuroxime, cefotiam, ceftazidime, ciprofloxacin, and imipenem. However, when cultured in MH medium containing ½ MIC gentamicin, the *yhjX*-deleted strain showed a higher growth rate than the wild-type and complemented strains, suggesting that *yhjX* contributes to bacterial tolerance to a subinhibitory concentration of gentamicin.

A previous study reported that *yhjX* of *E. coli* was highly activated in the presence of 1,4-butanediol; however, overexpression of the *yhjX* gene did not result in any improvement in 1,4-BDO tolerance (Szmidt-Middleton et al., 2013). In contrast, *yhjX* deletion improved the growth of *E. coli* strains in the control defined medium but not in 1,4-BDO (Szmidt-Middleton et al., 2013). Behr et al. (2014) reported that shortage of certain C sources increases extracellular pyruvate release and thereby triggers the expression of yhjX (Fried et al., 2013). However, whether *yhjX* inversely plays a role in pyruvate efflux and thereby decreases the growth of *E. coli* remains unclear. Our qRT-PCR data showed that relative to the control (grown in M9 minimal medium with glucose), the expression levels of *yhjX* in *E. coli* grown in MH medium, MH medium with gentamicin or M9 minimal medium were higher at both 30 and 60 min. A sharp increase in extracellular pyruvate levels at 30 min and a subsequent decrease at 60 min could be detected when wild-type *E. coli* was grown in MH medium, MHB containing 0.4% gentamicin or M9 containing 0.4% glucuronate. However, for the ΔyhjX knock-out mutant, the extracellular levels of pyruvate did not decrease significantly at 60 min after inoculation (compared to the level of pyruvate at 30 min) in any of the media. The above results suggested that *yhjX* might not encode a pyruvate efflux pump in *E. coli* but was associated with the decrease following a sharp increase in the extracellular pyruvate level. This finding was in consistent with the study reported by Fried et al. (2013). In addition, although *yhjX* expression was greatly induced in gentamicin-containing MH medium, we did not detect a marked change in extracellular pyruvate levels compared to the levels in MH medium without gentamicin. This result implies that pyruvate is not the only factor that triggers the induction of *yhjX* expression. Because the subinhibitory concentration of gentamicin influenced the metabolic pathway of *E. coli* by downregulating the TCA cycle enzymes, we hypothesize that the presence of a subinhibitory concentration of gentamicin induces *yhjX* expression by interfering with the nutrient and energy metabolism pathways of *E. coli*. *YhjX* was also predicted to be involved in the exchange carboxylic acids based on sequence similarity to the oxalate:formate antiporter OxlT in *Oxalobacter formigenes* (Pao et al., 1998; Keseler et al., 2009). In *O. formigenes*, oxalate is imported by OxlT to synthesize acetyl-CoA for further energy production. YhjX functioning as an oxalate transporter like OxlT might explain the higher extracellular pyruvate levels of the ΔyhjX mutant. Further studies are needed to investigate the specific function of YhjX and the mode of action of this protein.

To determine whether *yhjX* is associated with adaptive resistance to gentamicin, we monitored the growth of the Δ*yhjX* mutant during the second exposure to ½ MIC gentamicin. Unlike the *E. coli* wild-type strain and complemented strain, the Δ*yhjX* mutant during the second exposure to gentamicin showed a lower growth rate than the control (the Δ*yhjX* mutant during the first exposure to gentamicin), indicating that no adaptive resistance to gentamicin was induced in the Δ*yhjX* mutant. The complemented strain regained the adaptive resistance when exposed to gentamicin for a second time. These results imply that *yhjX* mediates the adaptive resistance of *E. coli* to subinhibitory concentrations of gentamicin.

*YhjX* is suggested to be the only target gene regulated by the YpdA/YpdB system of *E. coli* (Fried et al., 2013; Behr et al., 2017). *YhjX* expression is dependent on the specific binding of 6 × His-ypdB with the *yhjX* promoter (Fried et al., 2013). In this study, no change in the expression of ypdB in the presence of 1/2 MIC gentamicin was observed by qRT-PCR or transcriptome sequencing (data not shown), suggesting that the induction of *yhjX* expression by gentamicin might be mediated by post-transcriptional regulation of *ypdB*.

It has been reported that *yhjX* contributes to nutrient scavenging before cells enter the stationary phase. It seems that *yhjX* limits bacterial growth under specific stress conditions via the control of nutrient consumption. Taken together, our results indicate that *yhjX* facilitates a sensitive bacterial response to environmental stress. This protein also functions as a regulator of bacterial growth and metabolism in nutrient-limited or energy-scarce conditions. *YhjX* expression is highly upregulated by specific stress conditions (such as the TCA cycle-inhibited condition in the presence of gentamicin), which in turn reduces the growth and metabolism of bacteria. This response of bacteria may aid the long-term survival of the cells in a nutrient- or energy-limited environment. When the *yhjX* gene is deleted, bacteria lose the ability to regulate growth and metabolism under environmental stress. Therefore, the growth and metabolism of *yhjX*-deficient bacteria are accelerated under environmental stress, and simultaneously, the long-term tolerance to the stress condition is also impaired. This finding may explain the impaired ability of adaptive resistance of the *E. coli* Δ*yhjX* mutant during the second exposure to gentamicin. Further study is needed to investigate the underlying molecular mechanisms of the involvement of *yhjX* in bacterial growth and adaptive resistance. In addition, this study needs to be extended to the tests on clinical strains, not just limited to a standard strain.

## Conclusion

The function of *yhjX* in *E. coli* is complex, which may be associated with the regulation of bacterial growth under specific stress conditions. It also mediates the adaptive resistance of *E. coli* to subinhibitory concentrations of gentamicin.

## Author Contributions

SZ and YZ completed the majority of this study and contributed equally. SZ wrote the first draft. XJZ, FY, and HFL helped with the gene knockout experiments. HYL, XGZ, JW, HZ, YH, and SL assisted with the bacteria experiments. GN assisted with the other experiments. QP designed the experiments, provided funding, and revised the manuscript.

## Conflict of Interest Statement

The authors declare that the research was conducted in the absence of any commercial or financial relationships that could be construed as a potential conflict of interest.

## Footnotes

1 https://www.ncbi.nlm.nih.gov/gene/948066

2 https://www.kegg.jp/kegg/pathway.html
