# Supplementary material for: YhjX Regulates the Growth of Escherichia coli in the Presence of a Subinhibitory Concentration of Gentamicin and Mediates the Adaptive Resistance to Gentamicin
Source: Front Microbiol. 2019 May 27;10:1180. doi: 10.3389/fmicb.2019.01180 (PMC6545925; doi:10.3389/fmicb.2019.01180)
Supplement: Supplementary file 1 [file Table_1.DOCX]

Supplementary data

Table 1. Genes up-regulated by sub-MIC of gentamicin

| Gene | Description | | | | | | Locus | Fold-change | Role category |
| --- | --- | --- | --- | --- | --- | --- | --- | --- | --- |
| *yhjX* | membrane protein | | | | | | CP009072.1:1033854-1035062(+) | 20.65 | Membrane and transport |
| DR76_00370 | PTS fructose transporter subunit IIBC | | | | | | CP009072.1:80049-81740(-) | 15.26 | Membrane and transport |
| DR76_00380 | PTS fructose transporter subunit IIA | | | | | | CP009072.1:82695-83825(-) | 15.06 | Membrane and transport |
| *kdpF* | potassium transporter TrkA | | | | | | CP009072.1:4520365-4520454(+) | 14.95 | Membrane and transport |
| DR76_22970 | citrate:succinate antiporter | | | | | | CP009072.1:4602100-4603563(+) | 11.19 | Membrane and transport |
| DR76_22550 | potassium-transporting ATPase subunit A | | | | | | CP009072.1:4520454-4522127(+) | 9.18 | Membrane and transport |
| DR76_11985 | PTS maltose transporter subunit IIBC | | | | | | CP009072.1:2471325-2472743(-) | 7.38 | Membrane and transport |
| DR76_22555 | potassium-transporting ATPase B chain | | | | | | CP009072.1:4522150-4524198(+) | 6.47 | Membrane and transport |
| DR76_17320 | inner membrane-associated protein | | | | | | CP009072.1:3531927-3532022(-) | 6.21 | Membrane and transport |
| DR76_11995 | magnesium-transporting ATPase | | | | | | CP009072.1:2474191-2476887(+) | 5.13 | Membrane and transport |
| DR76_11000 | membrane protein | | | | | | CP009072.1:2269508-2269789(-) | 4.74 | Membrane and transport |
| *glnQ* | glutamine ABC transporter ATP-binding protein | | | | | | CP009072.1:4421108-4421830(+) | 4.13 | Membrane and transport |
| *artP* | arginine ABC transporter ATP-binding protein | | | | | | CP009072.1:4571958-4572683(+) | 3.37 | Membrane and transport |
| *fxsA* | exclusion suppressor FxsA | | | | | | CP009072.1:2367299-2367775(+) | 3.14 | Membrane and transport |
| Table1. ( continued ) | | | | | | | | | |
| Gene | Discription | | | | | | Locus | Fold-change | Role and category |
| DR76_06575 | membrane protein | | | | | | CP009072.1:1375524-1375787(-) | 3.10 | Membrane and transport |
| *glnP* | glutamine transporter subunit | | | | | | CP009072.1:4420452-4421111(+) | 3.00 | Membrane and transport |
| DR76_00230 | UPF0299 family inner membrane protein | | | | | | CP009072.1:52115-52513(+) | 2.52 | Membrane and transport |
| DR76_05890 | putative transporter | | | | | | CP009072.1:1259761-1260942(-) | 2.48 | Membrane and transport |
| *cysW* | sulfate/thiosulfate ABC transporter permease | | | | | | CP009072.1:362019-362894(-) | 2.46 | Membrane and transport |
| DR76_10140 | multidrug transporter | | | | | | CP009072.1:2090493-2091665(-) | 2.44 | Membrane and transport |
| DR76_01705 | sulfate/thiosulfate import ATP-binding protein CysA | | | | | | CP009072.1:360932-362029(-) | 2.43 | Membrane and transport |
| *malG* | maltose transporter subunit | | | | | | CP009072.1:2218708-2219598(-) | 2.40 | Membrane and transport |
| DR76_22800 | glutamate/aspartate ABC transporter permease | | | | | | CP009072.1:4571284-4571958(+) | 2.39 | Membrane and transport |
| *nhaA* | sodium:proton antiporter | | | | | | CP009072.1:2694697-2695863(+) | 2.32 | Membrane and transport |
| DR76_18795 | transporter | | | | | | CP009072.1:3837512-3839164(+) | 2.31 | Membrane and transport |
| DR76_21540 | formate channel | | | | | | CP009072.1:4317979-4318836(+) | 2.31 | Membrane and transport |
| *glnH* | glutamine transporter subunit | | | | | | CP009072.1:4419567-4420313(+) | 2.26 | Membrane and transport |
| DR76_16215 | membrane protein | | | | | | CP009072.1:3325585-3326175(-) | 2.20 | Membrane and transport |
| *secY* | preprotein translocase subunit SecY | | | | | | CP009072.1:1309425-1310756(+) | 2.15 | Membrane and transport |
| DR76_20395 | membrane protein | | | | | | CP009072.1:4083530-4084021(-) | 2.14 | Membrane and transport |
| DR76_22560 | potassium-transporting ATPase subunit C | | | | | | CP009072.1:4524207-4524779(+) | 2.14 | Membrane and transport |
|  | | | | | | | | | |
| Table 1(continued) | | |  | | | |  |  |  |
| Gene | | | Discription | | | | Locus | Fold-change | Role and category |
| *malF* | | | maltose transporter membrane protein | | | | CP009072.1:2219613-2221157(-) | 2.11 | Membrane and transport |
| DR76_06970 | | | membrane protein | | | | CP009072.1:1452429-1453469(+) | 2.09 | Membrane and transport |
| DR76_15700 | | | putative inner membrane protein | | | | CP009072.1:3231263-3231550(+) | 2.05 | Membrane and transport |
| DR76_18155 | | | membrane protein YcjF | | | | CP009072.1:3709681-3710742(-) | 2.01 | Membrane and transport |
| *fruK* | | | 1-phosphofructokinase | | | | CP009072.1:81757-82695(-) | 18.79 | Glycolysis / Gluconeogenesis |
| DR76_16485 | | | pyruvate kinase | | | | CP009072.1:3378231-3379643(-) | 4.76 | Glycolysis / Gluconeogenesis |
| DR76_04550 | | | 3-bisphosphoglycerate-independent phosphoglycerate mutase | | | | CP009072.1:963909-965453(-) | 4.36 | Glycolysis / Gluconeogenesis |
| DR76_02770 | | | 6-phosphofructokinase | | | | CP009072.1:605944-606906(-) | 2.75 | Glycolysis / Gluconeogenesis |
| *epd* | | | erythrose-4-phosphate dehydrogenase | | | | CP009072.1:1818728-1819747(+) | 2.63 | Glycolysis / Gluconeogenesis |
| DR76_18590 | | | acetaldehyde dehydrogenase | | | | CP009072.1:3794917-3797592(+) | 2.50 | Glycolysis / Gluconeogenesis |
| *RelE* | | | toxin RelE | | | | CP009073.1:15512-15859(+) | 4.98 | Virulence |
| DR76_08000 | | | capsular polysaccharide biosynthesis protein | | | | CP009072.1:1664756-1665994(-) | 2.46 | Virulence |
| DR76_17465 | | | fimbrial protein | | | | CP009072.1:3557959-3558522(+) | 14.26 | Motility |
|  | | |  | | | |  |  |  |
| Table 1(continued) | | |  | | | |  |  |  |
| Gene | | | Discription | | | | Locus | Fold-change | Role and category |
| DR76_23225 | | | tail fiber assembly protein | | | | CP009072.1:4661127-4661432(-) | 4.25 | Motility |
| *fliE* | | | flagellar hook-basal body protein FliE | | | | CP009072.1:3127418-3127732(+) | 3.31 | Motility |
| *csgC* | | | curli assembly protein CsgC | | | | CP009072.1:4079022-4079354(-) | 3.06 | Motility |
| *fliR* | | | flagellar biosynthesis protein FliR | | | | CP009072.1:3116753-3117538(-) | 2.99 | Motility |
| *fliG* | | | flagellar motor switch protein FliG | | | | CP009072.1:3124557-3125552(-) | 2.69 | Motility |
| *fliP* | | | flagellar biosynthesis protein FliP | | | | CP009072.1:3117826-3118563(-) | 2.60 | Motility |
| *fliH* | | | flagellar assembly protein H | | | | CP009072.1:3123878-3124564(-) | 2.58 | Motility |
| *fliQ* | | | flagellar biosynthesis protein FliQ | | | | CP009072.1:3117547-3117816(-) | 2.43 | Motility |
| *fliI* | | | ATP synthase | | | | CP009072.1:3122505-3123878(-) | 2.39 | Motility |
| DR76_15070 | | | flagellar biosynthesis protein FliO | | | | CP009072.1:3118563-3118928(-) | 2.36 | Motility |
| *fliN* | | | flagellar motor switch protein FliN | | | | CP009072.1:3118931-3119344(-) | 2.18 | Motility |
|  | | |  | | | |  |  |  |
| Table 1(continued) | | | | | | | | | |
| Gene | Discription | | | | | | Locus | Fold-change | Role and category |
| *fliF* | flagellar MS-ring protein | | | | | | CP009072.1:3125545-3127203(-) | 2.15 | Motility |
| *fliL* | flagellar protein FliL | | | | | | CP009072.1:3120350-3120814(-) | 2.14 | Motility |
| *fliJ* | flagellar biosynthesis chaperone | | | | | | CP009072.1:3122043-3122486(-) | 2.05 | Motility |
| *flgA* | flagellar basal body P-ring biosynthesis protein FlgA | | | | | | CP009072.1:4053451-4054110(+) | 2.04 | Motility |
| DR76_06145 | 50S ribosomal protein L22 | | | | | | CP009072.1:1303289-1303621(+) | 5.84 | Ribosome and translation |
| DR76_06140 | 30S ribosomal protein S19 | | | | | | CP009072.1:1302996-1303274(+) | 5.31 | Ribosome and translation |
| DR76_06150 | 30S ribosomal protein S3 | | | | | | CP009072.1:1303639-1304340(+) | 5.02 | Ribosome and translation |
| DR76_06135 | 50S ribosomal protein L2 | | | | | | CP009072.1:1302158-1302979(+) | 4.35 | Ribosome and translation |
| *prfH* | peptide chain release factor | | | | | | CP009072.1:4927383-4927997(-) | 4.17 | Ribosome and translation |
| DR76_03665 | tRNA-Trp | | | | | | CP009072.1:785950-786025(-) | 4.02 | Ribosome and translation |
| DR76_06165 | 30S ribosomal protein S17 | | | | | | CP009072.1:1304954-1305208(+) | 3.89 | Ribosome and translation |
| DR76_06155 | 50S ribosomal protein L16 | | | | | | CP009072.1:1304353-1304763(+) | 3.79 | Ribosome and translation |
| DR76_03670 | tRNA-Asp | | | | | | CP009072.1:786034-786110(-) | 3.79 | Ribosome and translation |
| DR76_06160 | 50S ribosomal protein L29 | | | | | | CP009072.1:1304763-1304954(+) | 3.74 | Ribosome and translation |
| *rplW* | 50S ribosomal protein L23 | | | | | | CP009072.1:1301838-1302140(+) | 3.64 | Ribosome and translation |
| DR76_10345 | 50S ribosomal protein L19 | | | | | | CP009072.1:2127979-2128326(+) | 3.59 | Ribosome and translation |
| DR76_06215 | 50S ribosomal protein L15 | | | | | | CP009072.1:1308983-1309417(+) | 3.51 | Ribosome and translation |
| *rpmD* | 50S ribosomal protein L30 | | | | | | CP009072.1:1308800-1308979(+) | 3.32 | Ribosome and translation |
| DR76_06205 | 30S ribosomal protein S5 | | | | | | CP009072.1:1308293-1308796(+) | 3.17 | Ribosome and translation |
| DR76_01575 | tRNA-Ala | | | | | | CP009072.1:340473-340548(-) | 2.95 | Ribosome and translation |
| DR76_06265 | putative regulator | | | | | | CP009072.1:1314873-1315091(+) | 2.95 | Ribosome and translation |
| Table 1(continued) | | | |  | | |  |  |  |
| Gene | | Discription | | | | | Locus | Fold-change | Role and category |
| *trmD* | | tRNA m(1)G37 methyltransferase | | | | | CP009072.1:2127170-2127937(+) | 2.89 | Ribosome and translation |
| *rplD* | | 50S ribosomal protein L4 | | | | | CP009072.1:1301236-1301841(+) | 2.83 | Ribosome and translation |
| *rplQ* | | 50S ribosomal protein L17 | | | | | CP009072.1:1313523-1313906(+) | 2.83 | Ribosome and translation |
| DR76_06195 | | 50S ribosomal protein L6 | | | | | CP009072.1:1307382-1307915(+) | 2.76 | Ribosome and translation |
| DR76_06200 | | 50S ribosomal protein L18 | | | | | CP009072.1:1307925-1308278(+) | 2.76 | Ribosome and translation |
| DR76_16540 | | tRNA-Val | | | | | CP009072.1:3388833-3388909(-) | 2.61 | Ribosome and translation |
| *truB* | | tRNA pseudouridine synthase B | | | | | CP009072.1:1438394-1439338(+) | 2.59 | Ribosome and translation |
| DR76_14680 | | tRNA-Asn | | | | | CP009072.1:3031903-3031978(+) | 2.56 | Ribosome and translation |
| DR76_13955 | | 30S ribosomal protein S2 | | | | | CP009072.1:2870600-2871325(+) | 2.55 | Ribosome and translation |
| DR76_12105 | | tRNA-Leu | | | | | CP009072.1:2499806-2499890(+) | 2.46 | Ribosome and translation |
| DR76_06120 | | 50S ribosomal protein L3 | | | | | CP009072.1:1300596-1301225(+) | 2.46 | Ribosome and translation |
| *rpsA* | | 30S ribosomal protein S1 | | | | | CP009072.1:4308776-4310449(-) | 2.45 | Ribosome and translation |
| DR76_06615 | | 30S ribosomal protein S9 | | | | | CP009072.1:1383118-1383510(+) | 2.44 | Ribosome and translation |
| *tsf* | | elongation factor Ts | | | | | CP009072.1:2871460-2872311(+) | 2.43 | Ribosome and translation |
| DR76_09465 | | tRNA-Met | | | | | CP009072.1:1961265-1961341(-) | 2.42 | Ribosome and translation |
| DR76_13355 | | 23S rRNA/tRNA pseudouridine synthase A | | | | | CP009072.1:2735354-2736013(-) | 2.42 | Ribosome and translation |
| *infB* | | translation initiation factor IF-2 | | | | | CP009072.1:1435157-1437829(+) | 2.42 | Ribosome and translation |
| DR76_09470 | | tRNA-Met | | | | | CP009072.1:1961375-1961451(-) | 2.41 | Ribosome and translation |
| DR76_09475 | | tRNA-Met | | | | | CP009072.1:1961485-1961561(-) | 2.40 | Ribosome and translation |
| DR76_01570 | | tRNA-Ala | | | | | CP009072.1:340358-340433(-) | 2.38 | Ribosome and translation |
| DR76_16535 | | tRNA-Val | | | | | CP009072.1:3388752-3388828(-) | 2.37 | Ribosome and translation |
| Table 1(continued) | | | | | |  |  |  |  |
| Gene | | Discription | | | | | Locus | Fold-change | Role and category |
| DR76_06190 | | 30S ribosomal protein S8 | | | | | CP009072.1:1306977-1307369(+) | 2.34 | Ribosome and translation |
| *rimM* | | ribosome maturation factor RimM | | | | | CP009072.1:2126591-2127139(+) | 2.32 | Ribosome and translation |
| *rbfA* | | ribosome-binding factor A | | | | | CP009072.1:1437993-1438394(+) | 2.30 | Ribosome and translation |
| DR76_01610 | | tRNA-Lys | | | | | CP009072.1:343568-343643(+) | 2.30 | Ribosome and translation |
| DR76_12860 | | tRNA-Leu | | | | | CP009072.1:2640600-2640686(-) | 2.29 | Ribosome and translation |
| DR76_1179 | | 30S ribosomal protein S10 | | | | | CP009072.1:1300252-1300563(+) | 2.28 | Ribosome and translation |
| DR76_10330 | | 30S ribosomal protein S16 | | | | | CP009072.1:2126324-2126572(+) | 2.26 | Ribosome and translation |
| *fusA* | | elongation factor G | | | | | CP009072.1:1279996-1282110(+) | 2.23 | Ribosome and translation |
| DR76_12855 | | tRNA-Leu | | | | | CP009072.1:2640485-2640571(-) | 2.19 | Ribosome and translation |
| DR76_10100 | | tRNA-Arg | | | | | CP009072.1:2083183-2083259(+) | 2.18 | Ribosome and translation |
| DR76_06005 | | sulfur transfer complex subunit TusB | | | | | CP009072.1:1278545-1278832(+) | 2.18 | Ribosome and translation |
| DR76_23240 | | tRNA-Arg | | | | | CP009072.1:4662125-4662201(-) | 2.17 | Ribosome and translation |
| DR76_14205 | | tRNA-Asp | | | | | CP009072.1:2918995-2919071(+) | 2.16 | Ribosome and translation |
| DR76_01595 | | tRNA-Val | | | | | CP009072.1:343247-343322(+) | 2.16 | Ribosome and translation |
| DR76_06610 | | 50S ribosomal protein L13 | | | | | CP009072.1:1382674-1383102(+) | 2.16 | Ribosome and translation |
| DR76_06240 | | 30S ribosomal protein S4 | | | | | CP009072.1:1311847-1312467(+) | 2.16 | Ribosome and translation |
| DR76_21630 | | tRNA-Ser | | | | | CP009072.1:4343304-4343391(+) | 2.15 | Ribosome and translation |
| DR76_01600 | | tRNA-Val | | | | | CP009072.1:343366-343441(+) | 2.15 | Ribosome and translation |
| DR76_12850 | | tRNA-Leu | | | | | CP009072.1:2640364-2640450(-) | 2.09 | Ribosome and translation |
| DR76_06870 | | tRNA-Met | | | | | CP009072.1:1432882-1432958(+) | 2.09 | Ribosome and translation |
| *rplI* | | 50S ribosomal protein L9 | | | | | CP009072.1:2423668-2424117(+) | 2.07 | Ribosome and translation |
| DR76_14800 | | tRNA-Asn | | | | | CP009072.1:3074202-3074277(-) | 2.07 | Ribosome and translation |
| Table 1(continued) | | | |  | | |  |  |  |
| Gene | | Discription | | | | | Locus | Fold-change | Role and category |
| *prfC* | | peptide chain release factor 3 | | | | | CP009072.1:2643722-2645311(+) | 2.04 | Ribosome and translation |
| DR76_10495 | | 50S ribosomal protein L1 | | | | | CP009072.1:2153316-2154020(+) | 2.02 | Ribosome and translation |
| DR76_01605 | | tRNA-Val | | | | | CP009072.1:343488-343563(+) | 2.02 | Ribosome and translation |
| DR76_06900 | | 30S ribosomal protein S15 | | | | | CP009072.1:1439487-1439756(+) | 2.01 | Ribosome and translation |
| DR76_03000 | | GTP-binding protein | | | | | CP009072.1:648503-650326(-) | 2.01 | Ribosome and translation |
| *rpsU* | | 30S ribosomal protein S21 | | | | | CP009072.1:1533209-1533424(-) | 2.00 | Ribosome and translation |
| DR76_17365 | | hypothetical protein | | | | | CP009072.1:3540708-3540926(-) | 13.14 | Hypothetical protein |
| DR76_19245 | | hypothetical protein | | | | | CP009072.1:3914999-3915292(+) | 11.75 | Hypothetical protein |
| DR76_17315 | | hypothetical protein | | | | | CP009072.1:3531748-3531927(-) | 5.27 | Hypothetical protein |
| DR76_10125 | | hypothetical protein | | | | | CP009072.1:2086874-2087986(-) | 4.34 | Hypothetical protein |
| DR76_25335 | | hypothetical protein | | | | | CP009072.1:5120964-5121095(-) | 3.95 | Hypothetical protein |
| DR76_25510 | | hypothetical protein | | | | | CP009073.1:16322-16702(+) | 3.73 | Hypothetical protein |
| DR76_25655 | | hypothetical protein | | | | | CP009073.1:32914-33111(+) | 3.73 | Hypothetical protein |
| DR76_25650 | | hypothetical protein | | | | | CP009073.1:32645-32914(+) | 3.56 | Hypothetical protein |
| DR76_25340 | | hypothetical protein | | | | | CP009072.1:5121271-5121603(-) | 3.51 | Hypothetical protein |
| DR76_01946 | | hypothetical protein | | | | | CP009072.1:2087983-2088680(-) | 3.42 | Hypothetical protein |
| DR76_19805 | | hypothetical protein | | | | | CP009072.1:3987261-3987437(+) | 3.42 | Hypothetical protein |
| DR76_10630 | | hypothetical protein | | | | | CP009072.1:2186608-2186991(+) | 3.10 | Hypothetical protein |
| DR76_06690 | | hypothetical protein | | | | | CP009072.1:1402229-1402861(-) | 3.04 | Hypothetical protein |
| DR76_18745 | | hypothetical protein | | | | | CP009072.1:3828983-3829120(+) | 2.97 | Hypothetical protein |
| DR76_15295 | | hypothetical protein | | | | | CP009072.1:3154267-3154590(-) | 2.88 | Hypothetical protein |
| DR76_04755 | | UPF0265 family protein | | | | | CP009072.1:5028629-5028958(-) | 2.72 | Hypothetical protein |
|  | |  | | | | |  |  |  |
| Table 1(continued) | | | |  | | |  |  |  |
| Gene | | Discription | | | | | Locus | Fold-change | Role and category |
| DR76_17825 | | hypothetical protein | | | | | CP009072.1:3637074-3637244(+) | 2.72 | Hypothetical protein |
| DR76_03025 | | uncharacterized protein | | | | | CP009072.1:654864-654974(+) | 2.69 | Hypothetical protein |
| DR76_03005 | | hypothetical protein | | | | | CP009072.1:650487-650720(+) | 2.65 | Hypothetical protein |
| DR76_01470 | | hypothetical protein | | | | | CP009072.1:318362-318637(+) | 2.60 | Hypothetical protein |
| DR76_01465 | | hypothetical protein | | | | | CP009072.1:317435-318067(-) | 2.57 | Hypothetical protein |
| DR76_24505 | | hypothetical protein | | | | | CP009072.1:4927994-4929133(-) | 2.56 | Hypothetical protein |
| DR76_25515 | | hypothetical protein | | | | | CP009073.1:16766-17038(+) | 2.54 | Hypothetical protein |
| DR76_12280 | | hypothetical protein | | | | | CP009072.1:2531199-2532116(+) | 2.54 | Hypothetical protein |
| DR76_22875 | | hypothetical protein | | | | | CP009072.1:4586640-4586903(+) | 2.53 | Hypothetical protein |
| DR76_14300 | | hypothetical protein | | | | | CP009072.1:2929800-2930033(-) | 2.43 | Hypothetical protein |
| DR76_20350 | | hypothetical protein | | | | | CP009072.1:4078590-4078901(-) | 2.40 | Hypothetical protein |
| DR76_25590 | | hypothetical protein | | | | | CP009073.1:26015-27082(+) | 2.37 | Hypothetical protein |
| DR76_17405 | | hypothetical protein | | | | | CP009072.1:3547588-3547947(+) | 2.31 | Hypothetical protein |
| DR76_12775 | | hypothetical protein | | | | | CP009072.1:2625595-2625717(-) | 2.21 | Hypothetical protein |
| DR76_21480 | | hypothetical protein | | | | | CP009072.1:4301825-4303057(-) | 2.20 | Hypothetical protein |
| DR76_20010 | | hypothetical protein | | | | | CP009072.1:4016165-4016704(-) | 2.01 | Hypothetical protein |
| *glnG* | | nitrogen regulation protein NR(I) | | | | | CP009072.1:653343-654752(+) | 4.11 | DNA binding and recombination |
| DR76_14230 | | transposase | | | | | CP009072.1:2922270-2922440(+) | 4.03 | DNA binding and recombination |
| *obgE* | | GTPase CgtA | | | | | CP009072.1:1419563-1420735(+) | 3.15 | DNA binding and recombination |
| DR76_06245 | | DNA-directed RNA polymerase subunit alpha | | | | | CP009072.1:1312493-1313482(+) | 2.63 | DNA binding and recombination |
| DR76_08135 | | transposase | | | | | CP009072.1:1688802-1690367(-) | 2.35 | DNA binding and recombination |
| Table 1(continued) | | | |  | | |  |  |  |
| Gene | | Discription | | | | | Locus | Fold-change | Role and category |
| DR76_21250 | | heat shock protein HspQ | | | | | CP009072.1:4250897-4251214(+) | 2.27 | DNA binding and recombination |
| DR76_21165 | | cold shock-like protein CspG | | | | | CP009072.1:4236992-4237204(-) | 2.24 | DNA binding and recombination |
| DR76_16210 | | L-cystine transporter tcyP | | | | | CP009072.1:3324061-3325452(-) | 2.22 | DNA binding and recombination |
| DR76_14795 | | integrase | | | | | CP009072.1:3072778-3074040(-) | 2.13 | DNA binding and recombination |
| DR76_21345 | | heme ABC transporter ATPase | | | | | CP009072.1:4267759-4269666(-) | 2.12 | DNA binding and recombination |
| DR76_24455 | | integrase | | | | | CP009072.1:4918921-4919259(-) | 2.07 | DNA binding and recombination |
| DR76_17595 | | putative DNA-binding transcriptional regulator | | | | | CP009072.1:3591490-3591774(+) | 2.06 | DNA binding and recombination |
| *zntR* | | zinc-responsive transcriptional regulator | | | | | CP009072.1:1314392-1314817(+) | 2.04 | DNA binding and recombination |
| *cpxP* | | inhibitor of the cpx response | | | | | CP009072.1:608138-608638(-) | 11.26 | Stress response |
| *pspG* | | phage-shock protein | | | | | CP009072.1:2235506-2235748(+) | 7.82 | Stress response |
| DR76_18245 | | phage-shock protein | | | | | CP009072.1:3727448-3728116(-) | 7.27 | Stress response |
| DR76_20000 | | multiple stress resistance protein BhsA | | | | | CP009072.1:4014973-4015230(-) | 6.39 | Stress response |
| DR76_18230 | | phage-shock protein | | | | | CP009072.1:3726581-3726802(-) | 5.83 | Stress response |
| DR76_04045 | | heat shock chaperone IbpB | | | | | CP009072.1:865383-865811(+) | 4.74 | Stress response |
| DR76_15675 | | heat shock protein HtpX | | | | | CP009072.1:3227235-3228116(+) | 4.34 | Stress response |
| DR76_04040 | | heat shock protein IbpA | | | | | CP009072.1:864858-865271(+) | 3.63 | Stress response |
| *emrB* | | multidrug resistance protein B | | | | | CP009072.1:2088938-2090476(-) | 3.22 | Stress response |
| Table 1(continued) | | | | |  | |  |  |  |
| Gene | | Discription | | | | | Locus | Fold-change | Role and category |
| *hslO* | | heat shock protein Hsp33 | | | | | CP009072.1:1228194-1229072(-) | 3.02 | Stress response |
| *glnL* | | nitrogen regulation protein NR(II) | | | | | CP009072.1:652282-653331(+) | 2.92 | Stress response |
| DR76_13125 | | molecular chaperone DnaJ | | | | | CP009072.1:2688798-2689928(+) | 2.92 | Stress response |
| DR76_17990 | | heat-inducible protein | | | | | CP009072.1:3679391-3679813(+) | 2.80 | Stress response |
| DR76_05740 | | ribosome-associated heat shock protein Hsp15 | | | | | CP009072.1:1229097-1229498(-) | 2.71 | Stress response |
| *nirD* | | nitrite reductase small subunit | | | | | CP009072.1:1256633-1256959(-) | 2.58 | Stress response |
| DR76_12770 | | carbon starvation protein CstA | | | | | CP009072.1:2623400-2625550(-) | 2.09 | Stress response |
| DR76_10310 | | co-chaperone GrpE | | | | | CP009072.1:2121913-2122506(+) | 2.08 | Stress response |
| DR76_00965 | | O-succinylbenzoic acid--CoA ligase | | | | | CP009072.1:201311-202666(-) | 2.01 | Biosynthesis of secondary metabolites |
| DR76_00970 | | O-succinylbenzoate synthase | | | | | CP009072.1:202663-203625(-) | 2.21 | Biosynthesis of secondary metabolites |
| DR76_02180 | | inositol monophosphatase | | | | | CP009072.1:472722-473525(+) | 2.31 | Biosynthesis of secondary metabolites |
| DR76_10195 | | hydrogen donor for NrdEF electron transport system | | | | | CP009072.1:2102133-2102378(-) | 4.29 | Electron carrier activity |
| DR76_21810 | | glutaredoxin | | | | | CP009072.1:4378275-4378532(+) | 2.46 | Electron carrier activity |
| DR76_09980 | | 4Fe-4S dicluster domain protein | | | | | CP009072.1:2062970-2063497(+) | 2.19 | Electron carrier activity |
|  | |  | | | | |  |  |  |
| Table 1(continued) | | | | |  | |  |  |  |
| Gene | | Discription | | | | | Locus | Fold-change | Role and category |
| DR76_09740 | | sulfite reductase subunit beta | | | | | CP009072.1:2019760-2021472(+) | 2.05 | Electron carrier activity |
| DR76_22945 | | citrate lyase subunit gamma | | | | | CP009072.1:4597897-4598193(+) | 18.11 | Carbohydrates metabolism |
| DR76_22950 | | citrate lyase, citryl-ACP lyase (beta) subunit | | | | | CP009072.1:4598190-4599098(+) | 10.92 | Carbohydrates metabolism |
| DR76_22940 | | citrate lyase ligase: [citrate [pro-3S]-lyase] ligase | | | | | CP009072.1:4596824-4597882(+) | 10.64 | Carbohydrates metabolism |
| DR76_22955 | | citrate lyase subunit alpha | | | | | CP009072.1:4599109-4600641(+) | 10.34 | Carbohydrates metabolism |
| DR76_25545 | | ATPase | | | | | CP009073.1:21388-22041(-) | 3.24 | Energy metobolism |
| DR76_09770 | | adenylylsulfate kinase | | | | | CP009072.1:2026448-2027053(+) | 2.21 | Energy metobolism |
| DR76_01130 | | acetate kinase | | | | | CP009072.1:237254-238456(+) | 2.05 | Energy metobolism |
| *citG* | | 2-(5''-triphosphoribosyl)-3'-dephosphocoenzyme-A synthase | | | | | CP009072.1:4601171-4602049(+) | 18.27 | Energy metobolism |
| *yneI* | | aldehyde dehydrogenase-like protein yneI | | | | | CP009072.1:3545209-3546114(+) | 3.21 | Energy metobolism |
| *citX* | | 2-(5''-triphosphoribosyl)-3'-dephospho-CoA synthase | | | | | CP009072.1:4600645-4601196(+) | 11.77 | Energy metobolism |
| DR76_23860 | | maltodextrin glucosidase | | | | | CP009072.1:4790160-4791974(-) | 6.34 | Starch and sucrose metabolism |
| DR76_20055 | | fused glucose-specific PTS enzymes: IIB component/IIC component | | | | | CP009072.1:4025017-4026450(-) | 6.29 | Starch and sucrose metabolism |
| *malQ* | | 4-alpha-glucanotransferase | | | | | CP009072.1:1208216-1210300(+) | 4.65 | Starch and sucrose metabolism |
|  | |  | | | | |  |  |  |
| Table 1(continued) | | | | | |  |  |  |  |
| Gene | | Discription | | | | | Locus | Fold-change | Role and category |
| DR76_05640 | | glycogen phosphorylase | | | | | CP009072.1:1205813-1208206(+) | 4.27 | Starch and sucrose metabolism |
| DR76_11980 | | trehalose-6-phosphate hydrolase | | | | | CP009072.1:2469620-2471275(-) | 4.04 | Starch and sucrose metabolism |
| *malS* | | alpha-amylase | | | | | CP009072.1:1007826-1009856(-) | 3.94 | Starch and sucrose metabolism |
| DR76_17370 | | transcriptional regulator | | | | | CP009072.1:3540958-3541347(-) | 10.36 | Trancription |
| DR76_17375 | | transcriptional regulator | | | | | CP009072.1:3541362-3541796(-) | 10.28 | Trancription |
| DR76_18235 | | transcriptional regulator | | | | | CP009072.1:3726811-3727170(-) | 5.81 | Trancription |
| *pspB* | | psp operon transcription co-activator | | | | | CP009072.1:3727170-3727394(-) | 5.48 | Trancription |
| DR76_25505 | | transcriptional regulator | | | | | CP009073.1:15859-16158(+) | 4.85 | Trancription |
| *dpiA* | | transcriptional regulatory protein DpiA | | | | | CP009072.1:4594137-4594817(-) | 3.03 | Trancription |
| *dpiB* | | sensor histidine kinase DpiB | | | | | CP009072.1:4594786-4596444(-) | 2.27 | Trancription |
| DR76_00620 | | transcriptional regulator | | | | | CP009072.1:119464-120111(+) | 2.04 | Trancription |
| DR76_09910 | | formate hydrogenlyase regulatory protein HycA | | | | | CP009072.1:2052515-2052976(+) | 2.01 | Trancription |
| *lpxK* | | tetraacyldisaccharide 4'-kinase | | | | | CP009072.1:4303094-4304080(-) | 2.52 | Lipid biosynthesis |
| DR76_02335 | | signal peptidase I | | | | | CP009072.1:508481-509455(-) | 2.29 | Protein and amino acid metabolism |
| DR76_23620 | | nitrogen assimilation regulatory protein for GlnL and AmtB | | | | | CP009072.1:4741683-4742021(-) | 3.45 | Amino acid biosynthesis |
| *glnA* | | glutamine synthetase | | | | | CP009072.1:650699-652108(+) | 2.77 | Amino acid biosynthesis |
|  | |  | | | | |  |  |  |
| Table 1(continued) | | | | | | | | | |
| Gene | | Discription | | | | | Locus | Fold-change | Role and category |
| DR76_06525 | | rod shape-determining protein MreD | | | | | CP009072.1:1362731-1363219(+) | 2.22 | Cell shape |
| DR76_22860 | | cell wall shape-determining protein | | | | | CP009072.1:4582968-4584080(+) | 2.00 | Cell shape |
| *pyrE* | | orotate phosphoribosyltransferase | | | | | CP009072.1:938132-938773(+) | 2.76 | Nucleic acid metabolism |
| DR76_05745 | | nucleotidase | | | | | CP009072.1:1229509-1230177(-) | 2.38 | Nucleic acid metabolism |
| *guaA* | | GMP synthase [glutamine-hydrolyzing] | | | | | CP009072.1:429203-430780(-) | 2.24 | Nucleic acid metabolism |
| DR76_16710 | | DNA glycosylase and apyrimidinic (AP) lyase (endonuclease III) | | | | | CP009072.1:3418795-3419430(-) | 2.16 | Nucleic acid metabolism |
| DR76_23155 | | dihydropteridine reductase | | | | | CP009072.1:4640270-4640923(+) | 2.04 | Nucleic acid metabolism |
| *dnaK* | | chaperone protein DnaK | | | | | CP009072.1:2686793-2688709(+) | 2.30 | DNA replication |
| DR76_24090 | | S-formylglutathione hydrolase | | | | | CP009072.1:4834808-4835641(+) | 2.20 | Methane metabolism |
