# Supplementary material for: YhjX Regulates the Growth of Escherichia coli in the Presence of a Subinhibitory Concentration of Gentamicin and Mediates the Adaptive Resistance to Gentamicin
Source: Front Microbiol. 2019 May 27;10:1180. doi: 10.3389/fmicb.2019.01180 (PMC6545925; doi:10.3389/fmicb.2019.01180)
Supplement: Supplementary file 2 [file Table_2.DOCX]

Supplementary data

Table 2. Genes down-regulated by sub-MIC of gentamicin

| Gene | Description | Locus | Fold-change | Role and category |
| --- | --- | --- | --- | --- |
| DR76_07080 | galactonate transporter | CP009072.1:1472578-1473912(+) | 0.08 | Membrane and transport |
| DR76_07215 | hexuronate transporter ExuT | CP009072.1:1498283-1499581(-) | 0.12 | Membrane and transport |
| *lacY* | lactose permease | CP009072.1:4840941-4842194(+) | 0.12 | Membrane and transport |
| DR76_07115 | threonine/serine transporter TdcC | CP009072.1:1480407-1481738(+) | 0.15 | Membrane and transport |
| DR76_05215 | membrane protein | CP009072.1:1114637-1115689(-) | 0.15 | Membrane and transport |
| DR76_17545 | antiporter | CP009072.1:3580658-3582193(+) | 0.17 | Membrane and transport |
| DR76_03920 | tryptophan permease | CP009072.1:840798-842045(-) | 0.17 | Membrane and transport |
| DR76_13265 | antiporter | CP009072.1:2716938-2718452(-) | 0.18 | Membrane and transport |
| DR76_09605 | glucarate transporter | CP009072.1:1986734-1988086(+) | 0.19 | Membrane and transport |
| DR76_12665 | nucleoside recognition pore and gate family putative inner membrane transporter | CP009072.1:2601335-2602018(-) | 0.20 | Membrane and transport |
| DR76_00275 | methyl-galactoside transporter subunit | CP009072.1:60841-61839(-) | 0.22 | Membrane and transport |
| DR76_00355 | nucleoside permease | CP009072.1:76382-77632(-) | 0.22 | Membrane and transport |
| DR76_05225 | multidrug ABC transporter ATP-binding protein | CP009072.1:1117487-1120222(+) | 0.23 | Membrane and transport |
| Table 2.（continued） |  |  |  |  |
| Gene | Description | Locus | Fold-change | Role and category |
| DR76_23440 | amino acid permease | CP009072.1:4700783-4702075(-) | 0.23 | Membrane and transport |
| DR76_20970 | membrane protein | CP009072.1:4192498-4194315(+) | 0.26 | Membrane and transport |
| *rbsC* | D-ribose ABC transporter permease | CP009072.1:796732-797697(-) | 0.27 | Membrane and transport |
| DR76_08895 | arginine exporter protein | CP009072.1:1823444-1824079(+) | 0.28 | Membrane and transport |
| *glpT* | sn-glycerol-3-phosphate transporter | CP009072.1:178850-180208(-) | 0.28 | Membrane and transport |
| DR76_11515 | outer membrane lipoprotein Blc | CP009072.1:2375511-2376044(-) | 0.29 | Membrane and transport |
| DR76_10365 | lipoprotein | CP009072.1:2130782-2131147(-) | 0.29 | Membrane and transport |
| *hdeB* | acid-resistance protein HdeB | CP009072.1:1089849-1090187(+) | 0.30 | Membrane and transport |
| DR76_10210 | putative membrane-anchored DUF883 family ribosome-binding protein | CP009072.1:2104496-2104825(-) | 0.31 | Membrane and transport |
| DR76_05230 | membrane protein | CP009072.1:1120222-1121346(+) | 0.31 | Membrane and transport |
| DR76_09845 | membrane protein | CP009072.1:2038888-2040150(-) | 0.31 | Membrane and transport |

| Table 2.（continued） |  |  |  |  |
| --- | --- | --- | --- | --- |
| Gene | Description | Locus | Fold-change | Role and category |
| DR76_02710 | glycerol uptake facilitator protein | CP009072.1:596332-597177(+) | 0.32 | Membrane and transport |
| DR76_03725 | D-ribose transporter ATP binding protein | CP009072.1:797702-799207(-) | 0.32 | Membrane and transport |
| DR76_16905 | membrane protein | CP009072.1:3459727-3460980(-) | 0.32 | Membrane and transport |
| DR76_00270 | D-ribose transporter ATP binding protein | CP009072.1:59260-60780(-) | 0.33 | Membrane and transport |
| DR76_14465 | membrane protein | CP009072.1:2957871-2958836(-) | 0.33 | Membrane and transport |
| DR76_04985 | C4-dicarboxylate ABC transporter | CP009072.1:1062728-1064014(+) | 0.34 | Membrane and transport |
| DR76_11750 | membrane protein | CP009072.1:2422075-2422350(-) | 0.34 | Membrane and transport |
| DR76_15855 | membrane protein | CP009072.1:3254994-3255242(+) | 0.35 | Membrane and transport |
| DR76_18580 | peptide ABC transporter substrate-binding protein | CP009072.1:3791424-3793055(-) | 0.35 | Membrane and transport |
| DR76_24160 | homoserine/Threonine efflux protein | CP009072.1:4853866-4854501(+) | 0.35 | Membrane and transport |
| DR76_15150 | outer membrane porin protein C | CP009072.1:3130438-3131520(-) | 0.36 | Membrane and transport |

| Table 2.（continued） |  |  |  |  |
| --- | --- | --- | --- | --- |
| Gene | Description | Locus | Fold-change | Role and category |
| DR76_05220 | membrane protein | CP009072.1:1116423-1117490(+) | 0.37 | Membrane and transport |
| DR76_04885 | peptide ABC transporter substrate-binding protein | CP009072.1:1037989-1039596(+) | 0.37 | Membrane and transport |
| DR76_00155 | MerR family transcriptional regulator | CP009072.1:36369-37100(+) | 0.37 | Membrane and transport |
| DR76_05020 | membrane protein | CP009072.1:1071778-1072791(-) | 0.38 | Membrane and transport |
| DR76_14945 | metal-binding protein ZinT | CP009072.1:3098536-3099186(-) | 0.38 | Membrane and transport |
| DR76_05085 | acid-resistance protein HdeA | CP009072.1:1089413-1089745(+) | 0.39 | Membrane and transport |
| DR76_00995 | protein ElaB | CP009072.1:208385-208690(-) | 0.39 | Membrane and transport |
| DR76_11920 | sugar ABC transporter substrate-binding protein | CP009072.1:2456500-2457456(+) | 0.40 | Membrane and transport |
| DR76_21725 | lipoprotein | CP009072.1:4364807-4365322(+) | 0.41 | Membrane and transport |
| DR76_05445 | membrane protein | CP009072.1:1159021-1159461(-) | 0.41 | Membrane and transport |
| DR76_14630 | multidrug transporter MatE | CP009072.1:3017284-3018723(+) | 0.43 | Membrane and transport |
| DR76_05065 | multidrug transporter | CP009072.1:1085764-1086921(-) | 0.43 | Membrane and transport |
| DR76_09875 | transporter | CP009072.1:2046061-2046489(-) | 0.44 | Membrane and transport |
| DR76_16955 | membrane protein | CP009072.1:3472809-3473114(-) | 0.44 | Membrane and transport |
| Table 2.（continued） |  |  |  |  |
| Gene | Description | Locus | Fold-change | Role and category |
| DR76_11505 | entericidin B membrane lipoprotein | CP009072.1:2374875-2375021(+) | 0.45 | Membrane and transport |
| DR76_05080 | acid-resistance protein HdeD | CP009072.1:1088586-1089158(-) | 0.45 | Membrane and transport |
| DR76_05150 | membrane protein | CP009072.1:1100642-1101208(-) | 0.46 | Membrane and transport |
| DR76_07230 | membrane protein | CP009072.1:1503061-1503612(+) | 0.46 | Membrane and transport |
| DR76_04940 | DUF2636 family cellulose production small membrane protein | CP009072.1:1047958-1048149(-) | 0.47 | Membrane and transport |
| DR76_20305 | SecY/SecA suppressor protein | CP009072.1:4070135-4070509(+) | 0.47 | Membrane and transport |
| DR76_05260 | PTS suar transporter subunit IIA | CP009072.1:1125812-1126285(-) | 0.47 | Membrane and transport |
| DR76_08655 | nucleoside permease | CP009072.1:1780987-1782243(-) | 0.48 | Membrane and transport |
| DR76_20235 | multidrug resistance protein MdtH | CP009072.1:4058988-4060196(+) | 0.48 | Membrane and transport |
| DR76_05095 | magnesium transporter ATPase | CP009072.1:1090251-1090898(+) | 0.49 | Membrane and transport |
| DR76_18510 | outer membrane protein W | CP009072.1:3779582-3780220(-) | 0.50 | Membrane and transport |
|  |  |  |  |  |
| Table 2.（continued） |  |  |  |  |
| Gene | Description | Locus | Fold-change | Role and category |
| DR76_01440 | histidine kinase | CP009072.1:308253-311636(+) | 0.50 | Membrane and transport |
| *gltA* | type II citrate synthase | CP009072.1:4509128-4510411(+) | 0.32 | TCA cycle |
| *sdhD* | succinate dehydrogenase cytochrome b556 small membrane subunit | CP009072.1:4507689-4508036(-) | 0.47 | TCA cycle |
| *sdhC* | succinate dehydrogenase cytochrome b556 large membrane subunit | CP009072.1:4508030-4508434(-) | 0.43 | TCA cycle |
| DR76_21695 | pyruvate dehydrogenase | CP009072.1:4358225-4359943(+) | 0.25 | TCA cycle |
| *aceE* | pyruvate dehydrogenase | CP009072.1:2804493-2807156(+) | 0.49 | TCA cycle |
| *sucC* | succinyl-CoA synthetase subunit beta | CP009072.1:4499347-4500513(-) | 0.48 | TCA cycle |
| DR76_19495 | isocitrate dehydrogenase | CP009072.1:3938376-3939626(-) | 0.34 | TCA cycle |
| DR76_16820 | fumarate hydratase class I | CP009072.1:3442314-3443960(+) | 0.44 | TCA cycle |
|  |  |  |  |  |
| Table 2.（continued） |  |  |  |  |
| Gene | Description | Locus | Fold-change | Role and category |
| DR76_04825 | XRE family transcriptional regulator | CP009072.1:1026093-1026383(-) | 0.22 | Transcription |
| DR76_07105 | transcriptional regulator | CP009072.1:1478359-1479297(+) | 0.27 | Transcription |
| DR76_05925 | transcriptional regulator | CP009072.1:1266758-1267390(-) | 0.42 | Transcription |
| DR76_10015 | transcriptional regulator | CP009072.1:2072191-2072550(-) | 0.44 | Transcription |
| DR76_13235 | transcriptional regulator | CP009072.1:2710790-2711185(+) | 0.45 | Transcription |
| DR76_20605 | transcriptional regulator | CP009072.1:4126838-4127338(-) | 0.50 | Transcription |
| DR76_01860 | transaldolase A | CP009072.1:390704-391654(+) | 0.26 | Transcription |
| DR76_02190 | stationary phase inducible protein CsiE | CP009072.1:474715-475995(+) | 0.26 | Transcription |
| DR76_05265 | regulatory protein | CP009072.1:1126310-1127056(-) | 0.49 | Transcription |
| *melR* | melibiose operon regulatory protein | CP009072.1:2341003-2341911(-) | 0.47 | Transcription |
| DR76_08905 | LysR family transcriptional regulator | CP009072.1:1825070-1825966(+) | 0.34 | Transcription |
|  |  |  |  |  |
| Table 2.（continued） |  |  |  |  |
| Gene | Description | Locus | Fold-change | Role and category |
| DR76_14570 | LuxR family transcriptional regulator | CP009072.1:2978294-2978506(-) | 0.18 | Transcription |
| DR76_01435 | LuxR family transcriptional regulator | CP009072.1:307424-308038(+) | 0.47 | Transcription |
| DR76_12445 | IclR family transcriptional regulator | CP009072.1:2563663-2564451(-) | 0.33 | Transcription |
| DR76_17760 | GntR family transcriptional regulator | CP009072.1:3627235-3627471(-) | 0.42 | Transcription |
| DR76_05075 | gad regulon transcriptional activator | CP009072.1:1087260-1087787(-) | 0.37 | Transcription |
| DR76_09145 | Fis family transcriptional regulator | CP009072.1:1880881-1882659(+) | 0.28 | Transcription |
| DR76_17560 | diguanylate phosphodiesterase | CP009072.1:3584186-3586609(+) | 0.45 | Transcription |
| DR76_05050 | AraC family transcriptional regulator | CP009072.1:1080342-1081166(+) | 0.41 | Transcription |
| DR76_07635 | AraC family transcriptional regulator | CP009072.1:1587160-1587642(+) | 0.48 | Transcription |
|  |  |  |  |  |
| Table 2.（continued） |  |  |  |  |
| Gene | Description | Locus | Fold-change | Role and category |
| DR76_00765 | acetoacetate metabolism regulatory protein AtoC | CP009072.1:149999-151384(+) | 0.36 | Transcription |
| DR76_02610 | catalase/hydroperoxidase HPI(I) | CP009072.1:571090-573270(-) | 0.43 | Stress response |
| DR76_22985 | universal stress protein G | CP009072.1:4605480-4605908(+) | 0.40 | Stress response |
| DR76_18010 | universal stress protein F | CP009072.1:3685514-3685948(+) | 0.21 | Stress response |
| DR76_18095 | universal stress protein E | CP009072.1:3698165-3699115(+) | 0.42 | Stress response |
| DR76_02730 | universal stress protein D | CP009072.1:600737-601165(-) | 0.26 | Stress response |
| DR76_15340 | universal stress protein C | CP009072.1:3162631-3163059(-) | 0.45 | Stress response |
| DR76_05195 | universal stress protein A | CP009072.1:1110395-1110829(-) | 0.46 | Stress response |
| DR76_19040 | two-component-system connector protein AriR | CP009072.1:3881739-3882005(-) | 0.23 | Stress response |
| DR76_18910 | SpoVR family protein | CP009072.1:3863496-3865028(+) | 0.15 | Stress response |
| DR76_07370 | L(+)-tartrate dehydratase subunit beta | CP009072.1:1536229-1536834(-) | 0.25 | Stress response |
|  |  |  |  |  |
| Table 2.（continued） |  |  |  |  |
| Gene | Description | Locus | Fold-change | Role and category |
| *sufA* | iron-sulfur cluster assembly scaffold protein | CP009072.1:3370956-3371324(+) | 0.44 | Stress response |
| DR76_03930 | tryptophanase leader peptide | CP009072.1:843773-843847(-) | 0.13 | Protein and amino acid metabolism |
| DR76_07110 | threonine dehydratase | CP009072.1:1479396-1480385(+) | 0.10 | Protein and amino acid metabolism |
| DR76_07315 | putrescine--2-oxoglutarate aminotransferase | CP009072.1:1523333-1524712(-) | 0.45 | Protein and amino acid metabolism |
| DR76_07130 | putative reactive intermediate deaminase | CP009072.1:1485314-1485703(+) | 0.27 | Protein and amino acid metabolism |
| DR76_09130 | putative peptidase | CP009072.1:1876692-1877903(-) | 0.10 | Protein and amino acid metabolism |
| DR76_18960 | protein YcgK precursor | CP009072.1:3872678-3873079(+) | 0.46 | Protein and amino acid metabolism |
| DR76_07120 | propionate/acetate kinase | CP009072.1:1481764-1482972(+) | 0.18 | Protein and amino acid metabolism |
| DR76_09125 | phenylhydantoinase | CP009072.1:1875253-1876638(-) | 0.15 | Protein and amino acid metabolism |
| DR76_22580 | ornithine decarboxylase | CP009072.1:4528819-4531017(+) | 0.27 | Protein and amino acid metabolism |
|  |  |  |  |  |
| Table 2.（continued） |  |  |  |  |
| Gene | Description | Locus | Fold-change | Role and category |
| DR76_09140 | ornithine carbamoyltransferase | CP009072.1:1879215-1880402(-) | 0.04 | Protein and amino acid metabolism |
| DR76_12030 | ornithine carbamoyltransferase | CP009072.1:2481253-2482257(-) | 0.35 | Protein and amino acid metabolism |
| DR76_11410 | lysine decarboxylase CadA | CP009072.1:2355104-2357251(-) | 0.30 | Protein and amino acid metabolism |
| DR76_07135 | L-serine dehydratase | CP009072.1:1485775-1487139(+) | 0.31 | Protein and amino acid metabolism |
| *tnaA* | L-cysteine desulfhydrase | CP009072.1:842137-843552(-) | 0.10 | Protein and amino acid metabolism |
| *ansB* | L-asparaginase | CP009072.1:1787229-1788275(+) | 0.29 | Protein and amino acid metabolism |
| DR76_16695 | glutathionine S-transferase | CP009072.1:3415971-3416576(-) | 0.46 | Protein and amino acid metabolism |
| DR76_23445 | glutaminase | CP009072.1:4702078-4703010(-) | 0.17 | Protein and amino acid metabolism |
| DR76_17540 | glutamate decarboxylase | CP009072.1:3579102-3580502(+) | 0.15 | Protein and amino acid metabolism |
| DR76_05045 | glutamate decarboxylase | CP009072.1:1078574-1079974(+) | 0.17 | Protein and amino acid metabolism |
| DR76_01420 | D-serine dehydratase | CP009072.1:302871-304199(+) | 0.42 | Protein and amino acid metabolism |
|  |  |  |  |  |
| Table 2.（continued） |  |  |  |  |
| Gene | Description | Locus | Fold-change | Role and category |
| DR76_18905 | D-amino acid dehydrogenase | CP009072.1:3861868-3863166(-) | 0.43 | Protein and amino acid metabolism |
| DR76_12035 | carbamate kinase | CP009072.1:2482281-2483213(-) | 0.21 | Protein and amino acid metabolism |
| DR76_09120 | carbamate kinase | CP009072.1:1874273-1875205(-) | 0.36 | Protein and amino acid metabolism |
| *aspA* | aspartate ammonia-lyase | CP009072.1:2365526-2366962(-) | 0.38 | Protein and amino acid metabolism |
| DR76_12040 | arginine deiminase | CP009072.1:2483224-2484444(-) | 0.28 | Protein and amino acid metabolism |
| DR76_14625 | amidase | CP009072.1:3015759-3017222(+) | 0.45 | Protein and amino acid metabolism |
| DR76_16460 | cysteine desulfurase activator complex subunit SufD | CP009072.1:3373551-3374822(+) | 0.40 | Protein and amino acid metabolism |
| DR76_09135 | diaminopropionate ammonia-lyase | CP009072.1:1877961-1879157(-) | 0.07 | Protein and amino acid metabolism |
| DR76_18900 | alanine racemase | CP009072.1:3860788-3861858(-) | 0.45 | Protein and amino acid metabolism |
| DR76_01825 | ethanolamine utilization protein | CP009072.1:384195-384488(-) | 0.44 | Protein and amino acid metabolism |
| DR76_14585 | 3-hydroxybutyryl-CoA dehydrogenase | CP009072.1:2991275-2992144(+) | 0.37 | Protein and amino acid metabolism |
| DR76_14600 | transacylase | CP009072.1:2993553-2994821(+) | 0.45 | Lipid metabolism |
| Table 2.（continued） |  |  |  |  |
| Gene | Description | Locus | Fold-change | Role and category |
| DR76_14650 | thioesterase | CP009072.1:3027250-3027819(+) | 0.44 | Lipid metabolism |
| DR76_17510 | sulfatase | CP009072.1:3568623-3570305(+) | 0.21 | Lipid metabolism |
| *glpC* | sn-glycerol-3-phosphate dehydrogenase subunit C | CP009072.1:183355-184545(+) | 0.22 | Lipid metabolism |
| *glpA* | sn-glycerol-3-phosphate dehydrogenase subunit A | CP009072.1:180481-182109(+) | 0.41 | Lipid metabolism |
| DR76_25345 | lipid kinase | CP009072.1:5122003-5122902(+) | 0.44 | Lipid metabolism |
| *glpQ* | glycerophosphodiester phosphodiesterase | CP009072.1:177769-178845(-) | 0.27 | Lipid metabolism |
| DR76_00870 | glycerol-3-phosphate dehydrogenase subunit B | CP009072.1:182099-183358(+) | 0.23 | Lipid metabolism |
| *glpD* | glycerol-3-phosphate dehydrogenase | CP009072.1:1194756-1196261(-) | 0.45 | Lipid metabolism |
| DR76_16785 | 7-alpha-hydroxysteroid dehydrogenase | CP009072.1:3432825-3433592(+) | 0.47 | Lipid metabolism |
| DR76_14595 | acyl-CoA dehydrogenase | CP009072.1:2992426-2993556(+) | 0.41 | Lipid metabolism |
|  |  |  |  |  |
| Table 2.（continued） |  |  |  |  |
| Gene | Description | Locus | Fold-change | Role and category |
| DR76_01260 | 3-oxoacyl-[acyl-carrier-protein] synthase I | CP009072.1:263466-264686(-) | 0.45 | Lipid metabolism |
| DR76_17565 | peroxiredoxin | CP009072.1:3586798-3587229(-) | 0.31 | Oxidation-reduction process |
| *dmsB* | dimethylsulfoxide reductase, chain B | CP009072.1:3467072-3467689(-) | 0.42 | Oxidation-reduction process |
| DR76_09850 | oxidoreductase | CP009072.1:2040147-2041055(-) | 0.16 | Oxidation-reduction process |
| DR76_16500 | oxidoreductase | CP009072.1:3381513-3383615(+) | 0.36 | Oxidation-reduction process |
| DR76_23780 | oxidoreductase | CP009072.1:4776370-4777344(+) | 0.37 | Oxidation-reduction process |
| DR76_08900 | oxidative stress defense protein | CP009072.1:1824172-1824912(+) | 0.29 | Oxidation-reduction process |
| DR76_21095 | NAD(P)H:quinone oxidoreductase | CP009072.1:4222029-4222625(+) | 0.40 | Oxidation-reduction process |
| DR76_00041 | short chain dehydrogenase family protein | CP009072.1:47982-48743(-) | 0.47 | Oxidation-reduction process |
| DR76_01160 | GSH-dependent disulfide bond oxidoreductase | CP009072.1:244405-245052(+) | 0.29 | Oxidation-reduction process |
| DR76_15310 | ferritin | CP009072.1:3155384-3155887(-) | 0.22 | Oxidation-reduction process |
| DR76_16510 | ferredoxin | CP009072.1:3384444-3384998(+) | 0.34 | Oxidation-reduction process |
| DR76_09720 | ferredoxin | CP009072.1:2015680-2015940(-) | 0.35 | Oxidation-reduction process |
| DR76_00400 | D-mannonate oxidoreductase | CP009072.1:86574-88040(+) | 0.38 | Oxidation-reduction process |
| DR76_16590 | superoxide dismutase | CP009072.1:3399369-3399950(-) | 0.47 | Oxidation-reduction process |
| DR76_16640 | superoxide dismutase | CP009072.1:3406298-3406819(+) | 0.46 | Oxidation-reduction process |
|  |  |  |  |  |
| Table 2.（continued） |  |  |  |  |
| Gene | Description | Locus | Fold-change | Role and category |
| *dkgA* | 2,5-diketo-D-gluconic acid reductase | CP009072.1:1605704-1606531(-) | 0.30 | Oxidation-reduction process |
| DR76_09155 | xanthine dehydrogenase subunit B | CP009072.1:1883175-1884053(-) | 0.31 | Nucleic acid metabolism |
| DR76_09160 | xanthine dehydrogenase subunit A | CP009072.1:1884064-1886361(-) | 0.37 | Nucleic acid metabolism |
| DR76_02500 | soluble pyridine nucleotide transhydrogenase | CP009072.1:543550-544950(+) | 0.49 | Nucleic acid metabolism |
| *rihB* | ribonucleoside hydrolase | CP009072.1:74546-75487(-) | 0.19 | Nucleic acid metabolism |
| DR76_06570 | putative barnase inhibitor | CP009072.1:1375197-1375469(+) | 0.38 | Nucleic acid metabolism |
| DR76_00360 | pseudouridine-5'-phosphate glycosidase | CP009072.1:77726-78664(-) | 0.16 | Nucleic acid metabolism |
| DR76_00365 | pseudouridine kinase | CP009072.1:78652-79593(-) | 0.14 | Nucleic acid metabolism |
| DR76_00260 | dihydropyrimidine dehydrogenase subunit B | CP009072.1:56756-57991(+) | 0.27 | Nucleic acid metabolism |
| DR76_00255 | dihydropyrimidine dehydrogenase subunit A | CP009072.1:55524-56762(+) | 0.27 | Nucleic acid metabolism |
| *cpdB* | 3'-nucleotidase | CP009072.1:2441232-2443175(-) | 0.46 | Nucleic acid metabolism |
|  |  |  |  |  |
| Table 2.（continued） |  |  |  |  |
| Gene | Description | Locus | Fold-change | Role and category |
| DR76_11865 | 3'-5'-bisphosphate nucleotidase | CP009072.1:2443365-2444105(+) | 0.40 | Nucleic acid metabolism |
| DR76_17625 | nitrite extrusion protein 2 | CP009072.1:3597980-3599368(+) | 0.31 | Nitrogen metabolism |
| DR76_18685 | nitrate reductase molybdenum cofactor assembly chaperone NarJ | CP009072.1:3813638-3814348(-) | 0.36 | Nitrogen metabolism |
| *napB* | nitrate reductase cytochrome C550 subunit | CP009072.1:127057-127506(-) | 0.39 | Nitrogen metabolism |
| *narI* | nitrate reductase A subunit gamma | CP009072.1:3812961-3813638(-) | 0.37 | Nitrogen metabolism |
| *narH* | nitrate reductase A subunit beta | CP009072.1:3814345-3815883(-) | 0.32 | Nitrogen metabolism |
| *fimA* | type-1 fimbrial protein subunit A | CP009072.1:4134652-4135194(-) | 0.28 | Motility |
|  |  |  |  |  |
| Table 2.（continued） |  |  |  |  |
| Gene | Description | Locus | Fold-change | Role and category |
| DR76_08465 | Major pilus subunit operon regulatory protein | CP009072.1:1745109-1745342(-) | 0.49 | Motility |
| DR76_08475 | Major pilu subunit operon regulatory protein papB | CP009072.1:1745747-1746061(+) | 0.32 | Motility |
| DR76_15195 | flagellin | CP009072.1:3138916-3140703(+) | 0.48 | Motility |
| DR76_18880 | flagellar brake protein YcgR | CP009072.1:3856539-3857273(+) | 0.39 | Motility |
| DR76_08480 | F7-2 fimbrial protein | CP009072.1:1746268-1746834(+) | 0.23 | Motility |
| DR76_19045 | two-component-system connector protein YmgA | CP009072.1:3882034-3882306(-) | 0.48 | Virulence |
| DR76_25830 | SagC family bacteriocin biosynthesis cyclodehydratase | CP009074.1:14219-15271(-) | 0.20 | Virulence |
| DR76_08990 | hemolysin | CP009072.1:1843817-1844476(+) | 0.31 | Virulence |
| DR76_17570 | biofilm-dependent modulation protein | CP009072.1:3587574-3587789(+) | 0.38 | Virulence |
| DR76_09100 | selenate reductase | CP009072.1:1867687-1870785(-) | 0.17 | Metal ion binding |
|  |  |  |  |  |
| Table 2.（continued） |  |  |  |  |
| Gene | Description | Locus | Fold-change | Role and category |
| DR76_13285 | putative 4Fe-4S ferredoxin-type protein | CP009072.1:2721957-2722244(+) | 0.18 | Metal ion binding |
| DR76_14975 | heat shock protein HSP31 | CP009072.1:3103876-3104727(-) | 0.25 | Metal ion binding |
| DR76_16945 | DmsA/YnfE family anaerobic dimethyl sulfoxide reductase | CP009072.1:3467700-3470096(-) | 0.44 | Metal ion binding |
| DR76_21570 | dimethyl sulfoxide reductase subunit B | CP009072.1:4325191-4325808(-) | 0.47 | Metal ion binding |
| DR76_16950 | dimethyl sulfoxide reductase subunit A | CP009072.1:3470184-3472610(-) | 0.38 | Metal ion binding |
| DR76_21575 | dimethyl sulfoxide reductase subunit A | CP009072.1:4325819-4328263(-) | 0.44 | Metal ion binding |
| DR76_09840 | aldolase | CP009072.1:2038253-2038891(-) | 0.36 | Metal ion binding |
| *garR* | tartronate semialdehyde reductase | CP009072.1:1474728-1475618(+) | 0.12 | Glyoxylate and dicarboxylate metabolism |
| DR76_07375 | tartrate dehydratase subunit alpha | CP009072.1:1536831-1537739(-) | 0.13 | Glyoxylate and dicarboxylate metabolism |
| DR76_07930 | malate synthase G | CP009072.1:1646334-1648505(+) | 0.30 | Glyoxylate and dicarboxylate metabolism |
| *fucO* | lactaldehyde reductase | CP009072.1:1975773-1976921(+) | 0.21 | Glyoxylate and dicarboxylate metabolism |
|  |  |  |  |  |
| Table 2.（continued） |  |  |  |  |
| Gene | Description | Locus | Fold-change | Role and category |
| *glcF* | glycolate oxidase | CP009072.1:1644680-1645903(+) | 0.24 | Glyoxylate and dicarboxylate metabolism |
| DR76_07095 | glycerate kinase | CP009072.1:1475715-1476860(+) | 0.25 | Glyoxylate and dicarboxylate metabolism |
| DR76_02875 | formate dehydrogenase subunit alpha | CP009072.1:625418-627832(+) | 0.48 | Glyoxylate and dicarboxylate metabolism |
| DR76_14555 | transposase | CP009072.1:2976563-2977042(-) | 0.44 | DNA binding and recombination |
| DR76_14560 | transposase | CP009072.1:2977291-2977413(-) | 0.45 | DNA binding and recombination |
| DR76_24975 | transposase | CP009072.1:5031808-5032134(-) | 0.48 | DNA binding and recombination |
| DR76_11690 | isovaleryl CoA dehydrogenase | CP009072.1:2411835-2413460(+) | 0.44 | DNA binding and recombination |
| *cspD* | inhibitor of DNA cold shock domain protein CspD | CP009072.1:4346685-4346909(+) | 0.25 | DNA binding and recombination |
| DR76_21110 | DNA-binding protein | CP009072.1:4224687-4225607(+) | 0.39 | DNA binding and recombination |
| DR76_21115 | chaperone modulatory protein CbpM | CP009072.1:4225607-4225912(+) | 0.34 | DNA binding and recombination |
| DR76_21990 | DNA protection during starvation protein | CP009072.1:4418660-4419163(+) | 0.48 | DNA binding and recombination |
| DR76_21105 | glucose-1-phosphatase/inositol phosphatase | CP009072.1:4222911-4224152(-) | 0.34 | Glycolysis / Gluconeogenesis |
| DR76_01865 | transketolase | CP009072.1:391674-393677(+) | 0.33 | Glycolysis / Gluconeogenesis |
|  |  |  |  |  |
| Table 2.（continued） |  |  |  |  |
| Gene | Description | Locus | Fold-change | Role and category |
| *glgA* | glycogen synthase | CP009072.1:1180881-1182314(+) | 0.45 | Glycolysis / Gluconeogenesis |
| DR76_16350 | phosphoenolpyruvate synthase | CP009072.1:3348402-3350780(+) | 0.13 | Glycolysis / Gluconeogenesis |
| *fbp* | fructose-1,6-bisphosphatase class 1 | CP009072.1:2461149-2462147(-) | 0.27 | Glycolysis / Gluconeogenesis |
| DR76_07055 | tagatose-bisphosphate aldolase | CP009072.1:1467291-1468571(-) | 0.18 | Galactose metabolism |
| DR76_07040 | PTS N-acetylgalactosamine transporter subunit IID | CP009072.1:1465137-1466015(-) | 0.25 | Galactose metabolism |
| DR76_07015 | PTS N-acetylgalactosamine transporter subunit IIB | CP009072.1:1460536-1461012(-) | 0.27 | Galactose metabolism |
| DR76_07050 | PTS N-acetylgalactosamine transporter subunit IIB | CP009072.1:1466795-1467268(-) | 0.32 | Galactose metabolism |
| *kbaY* | tag_bisphos_ald: class II aldolase, tagatose | CP009072.1:1461179-1462039(-) | 0.24 | Galactose metabolism |
|  |  |  |  |  |
| Table 2.（continued） |  |  |  |  |
| Gene | Description | Locus | Fold-change | Role and category |
| *lacZ* | beta-D-galactosidase | CP009072.1:4837815-4840889(+) | 0.15 | Galactose metabolism |
| DR76_11345 | alpha-galactosidase | CP009072.1:2342194-2343549(+) | 0.12 | Galactose metabolism |
| DR76_05530 | glycogen-debranching protein | CP009072.1:1177595-1179568(+) | 0.45 | Glycogen metabolic process |
| DR76_05525 | glycogen-branching protein | CP009072.1:1175412-1177598(+) | 0.46 | Glycogen metabolic process |
| DR76_05545 | glycogen phosphorylase | CP009072.1:1182333-1184780(+) | 0.48 | Glycogen metabolic process |
| *glgC* | glucose-1-phosphate adenylyltransferase | CP009072.1:1179586-1180881(+) | 0.39 | Glycogen metabolic process |
| *glpK* | glycerol kinase | CP009072.1:597200-598708(+) | 0.35 | Glycerol metabolic process |
|  |  |  |  |  |
| Table 2.（continued） |  |  |  |  |
| Gene | Description | Locus | Fold-change | Role and category |
| *gldA* | glycerol dehydrogenase | CP009072.1:568068-569171(+) | 0.44 | Glycerol metabolic process |
| DR76_10020 | sorbitol-6-phosphate dehydrogenase | CP009072.1:2072656-2073435(-) | 0.32 | Fructose and mannose metabolism |
| *srlA* | PTS sorbitol transporter subunit IIC | CP009072.1:2074777-2075340(-) | 0.23 | Fructose and mannose metabolism |
| *srlE* | PTS sorbitol transporter subunit IIB | CP009072.1:2073821-2074780(-) | 0.20 | Fructose and mannose metabolism |
| DR76_10025 | PTS sorbitol transporter subunit IIA | CP009072.1:2073439-2073810(-) | 0.32 | Fructose and mannose metabolism |
| DR76_07035 | PTS N-acetylgalactosamine transporter subunit IIA | CP009072.1:1464685-1465119(-) | 0.36 | Fructose and mannose metabolism |
|  |  |  |  |  |
| Table 2.（continued） |  |  |  |  |
| Gene | Description | Locus | Fold-change | Role and category |
| DR76_15035 | mannosyl-3-phosphoglycerate phosphatase | CP009072.1:3114105-3114920(-) | 0.31 | Fructose and mannose metabolism |
| DR76_09545 | fuculose phosphate aldolase | CP009072.1:1975098-1975745(+) | 0.23 | Fructose and mannose metabolism |
| DR76_16515 | thiosulfate reductase cytochrome B | CP009072.1:3384995-3385780(+) | 0.29 | Electron transfer activity |
| DR76_13280 | putative oxidoreductase | CP009072.1:2720674-2721960(+) | 0.15 | Electron transfer activity |
| DR76_13275 | protein fixB | CP009072.1:2719710-2720651(+) | 0.19 | Electron transfer activity |
| DR76_20240 | glutaredoxin | CP009072.1:4060260-4060907(+) | 0.48 | Electron transfer activity |
| DR76_13270 | electron transfer flavoprotein FixB | CP009072.1:2718925-2719695(+) | 0.18 | Electron transfer activity |
| DR76_05040 | cytochrome C peroxidase | CP009072.1:1076966-1078363(+) | 0.28 | Electron transfer activity |
| DR76_15335 | trehalose-6-phosphate synthase | CP009072.1:3161200-3162624(+) | 0.38 | Cell shape |
| DR76_15330 | trehalose-6-phosphate phosphatase | CP009072.1:3160425-3161225(+) | 0.26 | Cell shape |
|  |  |  |  |  |
| Table 2.（continued） |  |  |  |  |
| Gene | Description | Locus | Fold-change | Role and category |
| DR76_10245 | peptidoglycan-binding protein LysM | CP009072.1:2108183-2108632(+) | 0.23 | Cell shape |
| DR76_16475 | putative L,D-transpeptidase YnhG | CP009072.1:3376617-3377621(+) | 0.37 | Cell shape |
| DR76_14590 | D-alanine--poly(phosphoribitol) ligase | CP009072.1:2992174-2992422(+) | 0.36 | Cell shape |
| DR76_14610 | polyketide synthase | CP009072.1:2999715-3002747(+) | 0.39 | Biosynthesis of secondary metabolites |
| DR76_14580 | polyketide synthase | CP009072.1:2988665-2991265(+) | 0.49 | Biosynthesis of secondary metabolites |
| DR76_14640 | polyketide synthase | CP009072.1:3023118-3025577(+) | 0.50 | Biosynthesis of secondary metabolites |
| DR76_10545 | phosphomethylpyrimidine synthase ThiC | CP009072.1:2167600-2169495(-) | 0.47 | Biosynthesis of secondary metabolites |
| DR76_06650 | N-acetylmannosamine kinase | CP009072.1:1389293-1390168(+) | 0.46 | Biosynthesis of secondary metabolites |
| DR76_07220 | glucuronate isomerase | CP009072.1:1500064-1501476(+) | 0.15 | Biosynthesis of secondary metabolites |
| DR76_03230 | carboxymethylenebutenolidase | CP009072.1:697559-698368(+) | 0.35 | Biosynthesis of secondary metabolites |
|  |  |  |  |  |
| Table 2.（continued） |  |  |  |  |
| Gene | Description | Locus | Fold-change | Role and category |
| DR76_14565 | 4'-phosphopantetheinyl transferase | CP009072.1:2977559-2978293(-) | 0.31 | Biosynthesis of secondary metabolites |
| DR76_13255 | crotonobetainyl-CoA:carnitine CoA-transferase | CP009072.1:2714420-2715637(-) | 0.49 | Carnitine metabolic process |
| DR76_13245 | carnitinyl-CoA dehydratase | CP009072.1:2711900-2712685(-) | 0.37 | Carnitine metabolic process |
| DR76_13240 | carnitine operon protein CaiE | CP009072.1:2711304-2711894(-) | 0.45 | Carnitine metabolic process |
| DR76_14475 | ribokinase RbsK | CP009072.1:2959662-2960876(-) | 0.32 | Carbohydrate metabolism |
| DR76_24705 | ribokinase | CP009072.1:4984737-4985657(-) | 0.33 | Carbohydrate metabolism |
| DR76_11695 | DUF1471 family periplasmic protein | CP009072.1:2413577-2413852(-) | 0.44 | Carbohydrate metabolism |
| DR76_02585 | fructose-bisphosphate aldolase | CP009072.1:567395-568057(+) | 0.48 | carbohydrate metabolism |
| DR76_07125 | keto-acid formate acetyltransferase | CP009072.1:1483006-1485300(+) | 0.23 | carbohydrate metabolism |
| DR76_07225 | altronate hydrolase | CP009072.1:1501491-1502978(+) | 0.20 | Carbohydrate metabolism. |
| *gudD* | glucarate dehydratase | CP009072.1:1989449-1990789(+) | 0.27 | Ascorbate and aldarate metabolism |
| DR76_09610 | glucarate dehydratase | CP009072.1:1988088-1989428(+) | 0.47 | Ascorbate and aldarate metabolism |
| *garD* | galactarate dehydrogenase | CP009072.1:1470632-1472203(-) | 0.23 | Ascorbate and aldarate metabolism |
|  |  |  |  |  |
| Table 2.（continued） |  |  |  |  |
| Gene | Description | Locus | Fold-change | Role and category |
| DR76_07085 | alpha-dehydro-beta-deoxy-D-glucarate aldolase | CP009072.1:1473928-1474698(+) | 0.15 | Ascorbate and aldarate metabolism |
| DR76_09570 | hypothetical protein | CP009072.1:1981164-1982528(-) | 0.44 | Hypothetical protein |
| DR76_15040 | uncharacterized protein | CP009072.1:3115218-3115445(-) | 0.18 | Hypothetical protein |
| DR76_24365 | uncharacterized protein | CP009072.1:4901352-4901453(+) | 0.19 | Hypothetical protein |
| DR76_12900 | uncharacterized protein | CP009072.1:2646436-2646597(+) | 0.28 | Hypothetical protein |
| DR76_21100 | uncharacterized protein | CP009072.1:4222646-4222873(+) | 0.42 | Hypothetical protein |
| DR76_19285 | hypothetical protein | CP009072.1:3919342-3919536(-) | 0.25 | Hypothetical protein |
| DR76_21085 | hypothetical protein | CP009072.1:4221483-4221656(-) | 0.35 | Hypothetical protein |
| DR76_25305 | hypothetical protein | CP009072.1:5113750-5114082(+) | 0.17 | Hypothetical protein |
| DR76_09110 | hypothetical protein | CP009072.1:1871789-1872559(+) | 0.18 | Hypothetical protein |
| DR76_17535 | hypothetical protein | CP009072.1:3578948-3579091(+) | 0.19 | Hypothetical protein |
| DR76_05830 | hypothetical protein | CP009072.1:1247294-1247656(+) | 0.20 | Hypothetical protein |
| DR76_25825 | hypothetical protein | CP009074.1:13008-14222(-) | 0.23 | Hypothetical protein |
|  |  |  |  |  |
| Table 2.（continued） |  |  |  |  |
| Gene | Description | Locus | Fold-change | Role and category |
| DR76_09115 | hypothetical protein | CP009072.1:1872607-1874232(+) | 0.24 | Hypothetical protein |
| DR76_07630 | hypothetical protein | CP009072.1:1586715-1587107(+) | 0.25 | Hypothetical protein |
| DR76_25820 | hypothetical protein | CP009074.1:12208-13011(-) | 0.25 | Hypothetical protein |
| DR76_16345 | hypothetical protein | CP009072.1:3348181-3348348(+) | 0.26 | Hypothetical protein |
| DR76_21560 | hypothetical protein | CP009072.1:4323664-4324290(+) | 0.26 | Hypothetical protein |
| DR76_12895 | hypothetical protein | CP009072.1:2645704-2646309(+) | 0.27 | Hypothetical protein |
| DR76_11380 | hypothetical protein | CP009072.1:2350474-2350704(+) | 0.28 | Hypothetical protein |
| DR76_11385 | hypothetical protein | CP009072.1:2350716-2350988(+) | 0.29 | Hypothetical protein |
| DR76_17870 | hypothetical protein | CP009072.1:3645456-3645680(-) | 0.29 | Hypothetical protein |
| DR76_18040 | hypothetical protein | CP009072.1:3690741-3690914(-) | 0.31 | Hypothetical protein |
| DR76_04615 | hypothetical protein | CP009072.1:980846-981055(+) | 0.31 | Hypothetical protein |
| DR76_14930 | hypothetical protein | CP009072.1:3094534-3096354(-) | 0.32 | Hypothetical protein |
| DR76_00070 | hypothetical protein | CP009072.1:12861-13403(-) | 0.33 | Hypothetical protein |
|  |  |  |  |  |
| Table 2.（continued） |  |  |  |  |
| Gene | Description | Locus | Fold-change | Role and category |
| DR76_04160 | hypothetical protein | CP009072.1:888714-889007(-) | 0.33 | Hypothetical protein |
| DR76_14370 | hypothetical protein | CP009072.1:2943615-2944787(-) | 0.33 | Hypothetical protein |
| DR76_16520 | hypothetical protein | CP009072.1:3385784-3386596(+) | 0.34 | Hypothetical protein |
| DR76_02735 | hypothetical protein | CP009072.1:601192-601491(-) | 0.34 | Hypothetical protein |
| DR76_25815 | hypothetical protein | CP009074.1:10454-12223(-) | 0.35 | Hypothetical protein |
| DR76_08470 | hypothetical protein | CP009072.1:1745593-1745760(+) | 0.35 | Hypothetical protein |
| DR76_19905 | hypothetical protein | CP009072.1:3995426-3995620(-) | 0.36 | Hypothetical protein |
| DR76_21090 | hypothetical protein | CP009072.1:4221589-4221825(-) | 0.36 | Hypothetical protein |
| DR76_21010 | hypothetical protein | CP009072.1:4204172-4204960(-) | 0.37 | Hypothetical protein |
| DR76_08700 | hypothetical protein | CP009072.1:1788392-1789399(+) | 0.37 | Hypothetical protein |
| DR76_16495 | hypothetical protein | CP009072.1:3380866-3381492(+) | 0.38 | Hypothetical protein |
| DR76_12925 | hypothetical protein | CP009072.1:2651121-2651222(+) | 0.38 | Hypothetical protein |
| DR76_10850 | hypothetical protein | CP009072.1:2233455-2233664(+) | 0.39 | Hypothetical protein |
|  |  |  |  |  |
| Table 2.（continued） |  |  |  |  |
| Gene | Description | Locus | Fold-change | Role and category |
| DR76_12690 | hypothetical protein | CP009072.1:2605260-2606027(-) | 0.39 | Hypothetical protein |
| DR76_20545 | hypothetical protein | CP009072.1:4111556-4111900(+) | 0.39 | Hypothetical protein |
| DR76_09835 | hypothetical protein | CP009072.1:2037472-2038248(-) | 0.39 | Hypothetical protein |
| DR76_15795 | hypothetical protein | CP009072.1:3245935-3246024(+) | 0.41 | Hypothetical protein |
| DR76_22110 | hypothetical protein | CP009072.1:4442803-4443564(+) | 0.41 | Hypothetical protein |
| DR76_14470 | hypothetical protein | CP009072.1:2958890-2959648(-) | 0.41 | Hypothetical protein |
| DR76_18125 | hypothetical protein | CP009072.1:3703735-3704655(+) | 0.41 | Hypothetical protein |
| DR76_12820 | hypothetical protein | CP009072.1:2635907-2636380(-) | 0.42 | Hypothetical protein |
| DR76_12685 | hypothetical protein | CP009072.1:2605006-2605263(-) | 0.42 | Hypothetical protein |
| DR76_19420 | hypothetical protein | CP009072.1:3931640-3931819(+) | 0.42 | Hypothetical protein |
| DR76_09975 | hypothetical protein | CP009072.1:2062157-2062585(-) | 0.43 | Hypothetical protein |
| DR76_22490 | hypothetical protein | CP009072.1:4510559-4510801(+) | 0.43 | Hypothetical protein |
| DR76_14870 | hypothetical protein | CP009072.1:3083456-3084628(+) | 0.43 | Hypothetical protein |
|  |  |  |  |  |
| Table 2.（continued） |  |  |  |  |
| Gene | Description | Locus | Fold-change | Role and category |
| DR76_00850 | hypothetical protein | CP009072.1:176916-177566(-) | 0.43 | Hypothetical protein |
| DR76_22815 | hypothetical protein | CP009072.1:4573781-4574263(-) | 0.44 | Hypothetical protein |
| DR76_03900 | hypothetical protein | CP009072.1:837063-837629(-) | 0.44 | Hypothetical protein |
| DR76_22315 | hypothetical protein | CP009072.1:4483037-4483417(+) | 0.44 | Hypothetical protein |
| DR76_07605 | hypothetical protein | CP009072.1:1582981-1583460(+) | 0.45 | Hypothetical protein |
| DR76_16505 | hypothetical protein | CP009072.1:3383628-3384266(+) | 0.45 | Hypothetical protein |
| DR76_15875 | hypothetical protein | CP009072.1:3256994-3257341(-) | 0.45 | Hypothetical protein |
| DR76_17120 | hypothetical protein | CP009072.1:3496114-3496290(+) | 0.45 | Hypothetical protein |
| DR76_14605 | hypothetical protein | CP009072.1:2994869-2999665(+) | 0.47 | Hypothetical protein |
| DR76_22030 | hypothetical protein | CP009072.1:4425371-4425631(+) | 0.47 | Hypothetical protein |
| DR76_16140 | hypothetical protein | CP009072.1:3312013-3312498(+) | 0.48 | Hypothetical protein |
| DR76_13675 | hypothetical protein | CP009072.1:2802822-2803112(+) | 0.48 | Hypothetical protein |
| DR76_05930 | hypothetical protein | CP009072.1:1267397-1267612(-) | 0.49 | Hypothetical protein |
|  |  |  |  |  |
| Table 2.（continued） |  |  |  |  |
| Gene | Description | Locus | Fold-change | Role and category |
| DR76_08580 | hypothetical protein | CP009072.1:1766615-1766857(-) | 0.49 | Hypothetical protein |
| DR76_09255 | hypothetical protein | CP009072.1:1905593-1905787(-) | 0.49 | Hypothetical protein |
| DR76_05825 | hypothetical protein | CP009072.1:1246372-1247277(+) | 0.49 | Hypothetical protein |
| DR76_12695 | hypothetical protein | CP009072.1:2606037-2607188(-) | 0.49 | Hypothetical protein |
| DR76_11290 | hypothetical protein | CP009072.1:2329632-2330510(+) | 0.50 | Hypothetical protein |
| DR76_09500 | hypothetical protein | CP009072.1:1965641-1965868(+) | 0.30 | Hypothetical protein |
| DR76_07925 | hypothetical protein | CP009072.1:1645908-1646312(+) | 0.21 | Hypothetical protein |
| *rpmJ* | RpmJ-like protein | CP009072.1:4902081-4902221(+) | 0.41 | Ribosome and translation |
| DR76_21325 | ribosome modulation factor | CP009072.1:4263749-4263916(-) | 0.31 | Ribosome and translation |
| DR76_14615 | peptide synthetase | CP009072.1:3002791-3009291(+) | 0.42 | Ribosome and translation |
| DR76_14635 | peptide synthetase | CP009072.1:3018720-3023087(+) | 0.46 | Ribosome and translation |
| DR76_16340 | PEP synthetase regulatory protein | CP009072.1:3347236-3348069(-) | 0.36 | Ribosome and translation |
|  |  |  |  |  |
| Table 2.（continued） |  |  |  |  |
| Gene | Description | Locus | Fold-change | Role and category |
| DR76_24370 | 50S ribosomal protein L31 type | CP009072.1:4901818-4902081(+) | 0.44 | Ribosome and translation |
| *rpsV* | 30S ribosomal protein S22 | CP009072.1:3587891-3588028(+) | 0.23 | Ribosome and translation |
| DR76_23140 | gamma-glutamyl:cysteine ligase | CP009072.1:4638311-4639429(+) | 0.32 | Amino acid biosynthesis |
| DR76_22305 | phospho-2-dehydro-3-deoxyheptonate aldolase | CP009072.1:4481669-4482721(-) | 0.46 | Amino acid biosynthesis |
| DR76_24555 | C-lysozyme inhibitor | CP009072.1:4939691-4940137(-) | 0.43 | Lysozyme inhibitor activity |
| *lacA* | galactoside O-acetyltransferase | CP009072.1:4842260-4842871(+) | 0.14 | Lactose biosynthesis |
| DR76_07320 | aerotaxis receptor | CP009072.1:1525130-1526650(+) | 0.41 | Chemotaxis |
